# GNA13 suppresses proliferation of ER+ breast cancer cells via ERα dependent upregulation of the *MYC* oncogene

**DOI:** 10.1186/s13058-024-01866-x

**Published:** 2024-07-04

**Authors:** Lalitha Vaishnavi Subramanyan, Suhail Ahmed Kabeer Rasheed, Lijin Wang, Sujoy Ghosh, Michelle Shi Ning Ong, Manikandan Lakshmanan, Mei Wang, Patrick J. Casey

**Affiliations:** 1 Programme in Cancer and Stem Cell Biology, Duke-NUS Medical School, Singapore, Singapore; 2 Centre for Computational Biology and Program in Cardiovascular and Metabolic Disorders, Duke-NUS Medical School, Singapore, Singapore; 3 Bioinformatics and Computational Biology, Pennington Biomedical Research Center, Baton Rouge, LA 70808 USA; 4 Biopharma Innovations and Solutions, Institute of Molecular and Cell Biology, Singapore, Singapore; 5 Department of Biochemistry, National University of Singapore, Singapore, Singapore; 6 Dept. of Pharmacology and Cancer Biology, Duke University Medical Center, Durham, NC USA

## Abstract

*GNA13* (Gα13) is one of two alpha subunit members of the G12/13 family of heterotrimeric G-proteins which mediate signaling downstream of GPCRs. It is known to be essential for embryonic development and vasculogenesis and has been increasingly shown to be involved in mediating several steps of cancer progression. Recent studies found that Gα13 can function as an oncogene and contributes to progression and metastasis of multiple tumor types, including ovarian, head and neck and prostate cancers. In most cases, Gα12 and Gα13, as closely related α-subunits in the subfamily, have similar cellular roles. However, in recent years their differences in signaling and function have started to emerge. We previously identified that Gα13 drives invasion of Triple Negative Breast Cancer (TNBC) cells in vitro. As a highly heterogenous disease with various well-defined molecular subtypes (ER+ /Her2−, ER+ /Her2+, Her2+, TNBC) and subtype associated outcomes, the function(s) of Gα13 beyond TNBC should be explored. Here, we report the finding that low expression of *GNA13* is predictive of poorer survival in breast cancer, which challenges the conventional idea of Gα12/13 being universal oncogenes in solid tumors. Consistently, we found that Gα13 suppresses the proliferation in multiple ER+ breast cancer cell lines (MCF-7, ZR-75-1 and T47D). Loss of *GNA13* expression drives cell proliferation, soft-agar colony formation and in vivo tumor formation in an orthotopic xenograft model. To evaluate the mechanism of Gα13 action, we performed RNA-sequencing analysis on these cell lines and found that loss of *GNA13* results in the upregulation of MYC signaling pathways in ER+  breast cancer cells. Simultaneous silencing of *MYC* reversed the proliferative effect from the loss of *GNA13*, validating the role of MYC in Gα13 regulation of proliferation. Further, we found Gα13 regulates the expression of MYC, at both the transcript and protein level in an ERα dependent manner. Taken together, our study provides the first evidence for a tumor suppressive role for Gα13 in breast cancer cells and demonstrates for the first time the direct involvement of Gα13 in ER-dependent regulation of MYC signaling. With a few exceptions, elevated Gα13 levels are generally considered to be oncogenic, similar to Gα12. This study demonstrates an unexpected tumor suppressive role for Gα13 in ER+ breast cancer via regulation of MYC, suggesting that Gα13 can have subtype-dependent tumor suppressive roles in breast cancer.

**Supplementary Information:**

The online version contains supplementary material available at 10.1186/s13058-024-01866-x.

## Introduction

Breast cancer (BrCa) is the most commonly diagnosed cancer and the leading cause of cancer-related-deaths in women worldwide, accounting for nearly 685,000 deaths in 2020 [1]. BrCa is a heterogeneous disease with diverse pathogenesis, which is mainly classified into four molecular subtypes, characterized by the expression of Estrogen Receptor (ERα), Progesterone receptor (PR) and the Epidermal Growth Factor Receptor (EGFR) family member Her2 [2]. These distinct subtypes of breast cancers are different in the molecular mechanisms that drive cancer progression and survival, with each of the subtypes displaying unique therapeutic vulnerabilities. Even within each subtype, there are often differences in molecular signature and pathogenesis making the targeted treatment a major challenge [3]. Hence, understanding subtype- and even tumor-specific mechanisms of breast cancer tumorigenesis and progression remains a cornerstone in developing suitable and most effective therapeutic regimens.

Nearly 80% of all breast cancers are positive for ERα (ER+), which accounts for the majority of the disease prevalence and disease burden. Endocrine therapy targeting ERα signaling remains the most widely applied therapeutic regimen in the clinic for this subtype [4]. Despite significant improvements in endocrine therapy over the past several decades, development of resistance remains a major concern [5], with nearly 50% of all patients eventually developing endocrine-resistant disease [6]. Hence it is imperative that the molecular mechanisms of resistance need to be further defined to improve disease outcome for majority of breast cancer patients [7].

G-protein coupled receptors (GPCRs), characterized by their 7 transmembrane (7TM) domains, belong to the largest known class of cell surface receptors with nearly 900 members expressed in humans [8]. GPCRs are involved in many aspects of normal physiology, including embryonic development, vision, immune signaling and metabolism [9–11]. Although they comprise the largest class of drug targets due to their involvement in a wide spectrum of biological functions and cell surface localization, GPCR targeting in cancer has significantly lagged. This is despite emerging evidence indicating the importance of several GPCRs—including CXCR4, Lysophosphatidic acid receptor (LPAR), Sphingosine-1-Phosphate Receptor (S1PR) and LGR5—in cancer [12]. In this regard, better understanding the specific roles of different GPCRs, G-proteins and their downstream signaling pathways would provide a promising avenue of cancer drug development.

Interestingly, many of the GPCRs implicated in cancer progression couple to the G12 subfamily. The G12 subfamily is comprised of two members, defined by their α-subunits—*GNA12* (Gα12) and *GNA13* (Gα13), that upon activation by GPCRs, appear to mainly function by activating Rho GTPases downstream [13, 14]. This Gα12/13-RhoA signaling has been implicated in the process of invasion and metastasis in multiple cancer models including those of breast (particularly TNBC), ovarian and prostate [15–19]. Further, Gα13 has also been shown to play a role in promoting cell proliferation [20], cancer cell stemness [12], and chemokine secretion [21]. To date, the study of function of Gα13 in breast cancer has been limited to TNBC cell models [15, 22, 23], and its role in other subtypes of breast cancer remain unknown.

We aimed to explore the role of Gα13 in other subtypes of breast cancers, particularly ER+ subtypes. Interestingly, we find that low *GNA13* expression predicts poorer overall survival in human breast cancer patients, which is somewhat surprising as both Gα12 and Gα13 are considered to promote tumorigenesis and cancer progression. In this study, we have focused on ER+ breast cancer cells, as they constitute ~ 80% of all breast cancers. Through cell proliferation and soft agar colony formation assays, we found that Gα13 indeed negatively regulates cell proliferation in ER+ breast cancer cells, but not in other subtypes of breast cancer cells. Further evaluations in ER+ breast cancer cells demonstrate that Gα13 controls cell proliferation by negatively regulating the expression of *MYC* oncogene and MYC signaling. Finally, we find that this regulation of MYC levels by Gα13 in ER+ BrCa cells is dependent on estrogen signaling. Considering that upregulation of *MYC* is one of the well-known mechanisms by which ER+ breast cancers acquire endocrine therapy resistance, our study points to a possible role for G-proteins in mediating endocrine therapy response.

## Materials and methods

### Cell lines and culture conditions and reagents

MCF-7, T47D, BT-474, MDA-MB-157, MDA-MB-436, MDA-MB-231, SKBR3, ZR-75-1, HCC-1428, CAMA-1 and HEK293T cells were grown in DMEM supplemented with 10% Fetal Bovine Serum (FBS) and 1% Penicillin/Streptomycin antibiotics. For MCF-7 and T47D cells, 10 µg/mL Human Insulin was also added to the medium. MDA-MB-134-VI, MDA-MB-361, UACC-812 cells were grown in Dulbecco’s Modified Eagle Medium (DMEM) supplemented with 20% Fetal Bovine Serum (FBS) and 1% Penicillin/Streptomycin. For drug treatments, fulvestrant and 17-β-Estradiol (E2) were purchased from Medchemexpress (HY-13636-10mM) and Sigma-Aldrich (E8875), respectively. For Estradiol deprivation condition, the DMEM (GIBCO, 31053028) was supplemented with 10% FBS pre-treated by Charcoal (GIBCO, 12676029), Sodium Pyruvate (GIBCO, 11360070) and GlutaMAX-I (GIBCO, 35050061) for indicated time periods. All cells were maintained in a 37 °C incubator with 5% CO_2_. All cell lines were authenticated by Short Tandem Repeat analysis (ATCC) and regularly tested for mycoplasma using the ABMGood® mycoplasma detection kit (# G238).

### Molecular biology

Short hairpin RNAs against GNA13 and the doxycycline-inducible GNA13 expression vector were produced as previously described [21]. Full-length GNA13 was amplified by PCR from PCDNA3.1-GNA13 construct and inserted into pLVX-CMV-puro construct (Clontech, # 632164) using Infusion Cloning kit (Clontech, # 638910) and confirmed by DNA sequencing. Details of primers used are provided in Supplementary Table 1.

### Stable cell line generation

MCF-7 and ZR-75-1 Cells with GNA13 stably silenced were obtained by retroviral transduction of shRNA into the cells and subsequent selection culturing with Blasticidin S hydrochloride. T47D, SKBR3 and MDA-MB-231 cells stably overexpressing GNA13 were obtained by lentiviral transduction of pLVX-CMV-puro-GNA13 or vector and subsequent selection by culturing in Puromycin. For rescue of GNA13 expression, MCF-7 cells expressing sh-Ctrl or shRNA against GNA13 were successively transduced with TET-3G and TRE-3G vec/GNA13 constructs as previously described and selected using G418 and Puromycin to obtain MCF-7 rescue cells.

### RNA isolation and quantitative RT-PCR

Total RNA was isolated using RNeasy mini-kit (Qiagen, # 74106) following manufacturers protocol; 1 µg of total RNA was used for cDNA synthesis using iScript Reverse Transcription kit (Bio-Rad, # 1708841). cDNA was diluted five-fold for downstream use. Quantitative RT-PCR was performed in triplicate using iQ SYBR green master mix (Bio-Rad, # 170-8880) and the Bio-Rad CFX 96/384 system. HPRT was used as loading control. Relative expression of mRNA was analyzed using ddCt method. Details of the primers used are given in Supplementary Table 2.

### RNA interference

Small interfering RNA (siRNA) targeting MYC and ESR1 were transfected into cells using the JetPrime Plus® transfection system (MCF-7 cells) or Lipofectamine RNAiMAX (MCF-7 and ZR-75-1 cells) at a final concentration of 100 nM according to manufacturer’s protocol. Cells were harvested after 48 h and subjected to experimental protocols as described in the appropriate section of Results. Details of siRNAs used are provided in supplementary Table 3.

### Immunoblotting

For protein lysate preparation, cells were first washed with PBS and lysed with Tris Lysis Buffer (1M tris buffer, pH 6.8, 2M NaCl, 1M MgCl_2_, 0.5M EDTA, 100nM EGTA, triton-X and glycerol) supplemented with protease inhibitor (Roche, 05892791001) and phosphatase inhibitor (Roche, 4906837001) Protein was quantified using BCA protein quantification kit (Thermo scientific, 23227); Laemmlli sample buffer with beta-mercaptoethanol was then added and samples were heated at 95 °C for 5–10 min. For immunoblot analysis, proteins were separated on SDS-PAGE gels and transferred onto PVDF membranes using wet transfer, the membranes are blocked with 5% Non-fat dry milk at room temperature for 1 h and incubated overnight in appropriate primary antibody overnight at 4 °C. Blots were then washed with Tris Buffered Saline-0.01% Tween-20 (TBS-T), incubated in respective secondary antibody for 1 h, washed again and imaged using Bio-Rad chemidoc MP imaging system using either Pierce® ECL Western Blotting Substrate (Cat. No.32106), ThermoFisher Scientific SuperSignal™ West Femto Maximum Sensitivity Substrate, (Cat. No. 34096), or Immobilon Forte western HRP substrate (Millipore, #WBLUF0500.) Antibody details are given in supplementary Table 4.

### 2D proliferation assays

To assess growth on plastic, 2500 and 5000 cells (3500 and 7000 cells for T47D) were seeded in 96-well plates and incubated for the indicated times. For cell viability measurements, 0.5X cell-titer glo (Promega, G7571) was added to the wells and the cells were allowed to lyse for 10 min at room temp on an orbital shaker, then, the lysate was transferred to white assay plates (Costar #3917); luminescence was measured using the TECAN multimodal imaging system. For live cell imaging assays, cells were seeded similarly in 96-well plates and imaged at regular time intervals using the IncuCyte ZOOM imaging platform, cell growth was measured as increase in confluence over time. For rescue experiments, protein expression was induced with 100 ng/mL of Doxycycline for 48 h prior to seeding, and doxycycline was replenished every alternate day to maintain GNA13 expression.

### Anchorage-independent growth assay

To assess anchorage-independent growth, 1 mL of a 1:1 mixture of 1.2% Agarose and 2X RPMI-1640 media supplemented with 20% FBS (and 20 ug/mL of insulin for MCF-7 and T47D) was aliquoted into 24-well Ultra-low Adherent plates (Corning, 3473) and allowed to solidify for at least 30 min. Then, cells were trypsinized, counted and resuspended in 1:1 mixture of 2X RPMI-1640 media, and 0.6% agarose then laid on top of the bottom agar layer. The gel was allowed to solidify for 20–30 min, whereupon 300 uL media was added on top to each well. Media was changed thrice weekly. After 21 days of growth, the colonies were stained with 100 uL of 5 mg/mL MTT dye per well for 1–4 h in 37 °C, imaged and colonies were counted using the Gel-count imaging system. For the rescue experiments, cells were treated with 100 ng/mL of Doxycycline for 48 h prior to seeding to induce GNA13 expression and maintained in 100 ng/mL of Doxycycline throughout the course of the experiment.

### RNA sequencing and analysis

MCF-7 stably expressing control sh-RNA or sh-RNAs directed against GNA13, and T47D, MDA-MB-231, SKBR3 cells stably overexpressing vector or GNA13, were seeded in 10-cm dishes and cultured until the cells reached 80% confluency. Total RNA was extracted for sequencing. Stranded mRNA library preparation and sequencing were done on the Novaseq 150PE platform (60M reads per sample). The RNA sequencing was performed by Biobasic (Singapore). The raw read files were checked for sequencing quality via FastQC program (https://www.bioinformatics.babraham.ac.uk/projects/fastqc/) and subsequently mapped to the human reference genome (GRCh38) via the STAR v2.7.7a aligner [24]. Mapped reads were quantified for gene and transcript abundance estimation via Rsubread v2.4.2 [25]. and further analyzed for differential gene expression via limma v3.4.2.2 [26]. Pathway level enrichment analysis was performed using Gene Set Enrichment Analysis (GSEA) tool (https://www.gsea-msigdb.org/gsea/index.jsp).

### Xenograft studies

All the animal experiments were carried out by animal care and use guidelines approved by the Biological Resource Centre, Singapore (IACUC # 231,783). MCF7 cells (5 × 10^6^ in 100 µl of DMEM) expressing either vector alone or sh-GNA13 were mixed with Corning® Matrigel® (Cat. No. 354234) in a 1:1 ratio were injected into the mammary fat pad of the female NOD-SCID mice (In Vivos, Singapore) (n = 5) respectively. All mice were monitored for tumor growth at the inoculation site and tumor. On Day 67, the tumors were harvested, and the tumor weights were measured using a weighing scale.

### Statistical analysis

All experiments were performed at least three times, and each with three or four technical replicates. Pooled data from at least three independent experiments is shown unless otherwise noted, and the p values presented are calculated from pooled values from three independent biological replicates. Data visualization and Statistical analysis was carried out using GraphPad Prism software (GraphPad, La Jolla, CA). Statistical significance was determined by Student’s unpaired t-test, one-way ANOVA, or two-way ANOVA.

## Results

### Gα13 expression varies in breast cancer cells; higher expression predicts poorer survival

In recent years, there has been an increasing interest in understanding the role of Gα13 in cancer [12, 21, 27, 28]. Up to now, the protein has been shown to be upregulated in more aggressive types of solid cancers, and its levels correlate to poorer prognosis [12, 15, 18, 29]. Gα13 has also been shown to induce migration, invasion and stemness when expressed in cancer cells [30–32]. In this vein, our previous studies in TNBC cells indicated that Gα13 contributes to increased migration, invasion and suppression of kallikreins (KLKs) in vitro [22, 23]. Here, we broadened the scope and examined the expression of Gα13 across subtypes in breast cancer and the impact of Gα13 expression level on patient survival.

**Fig. 1:**
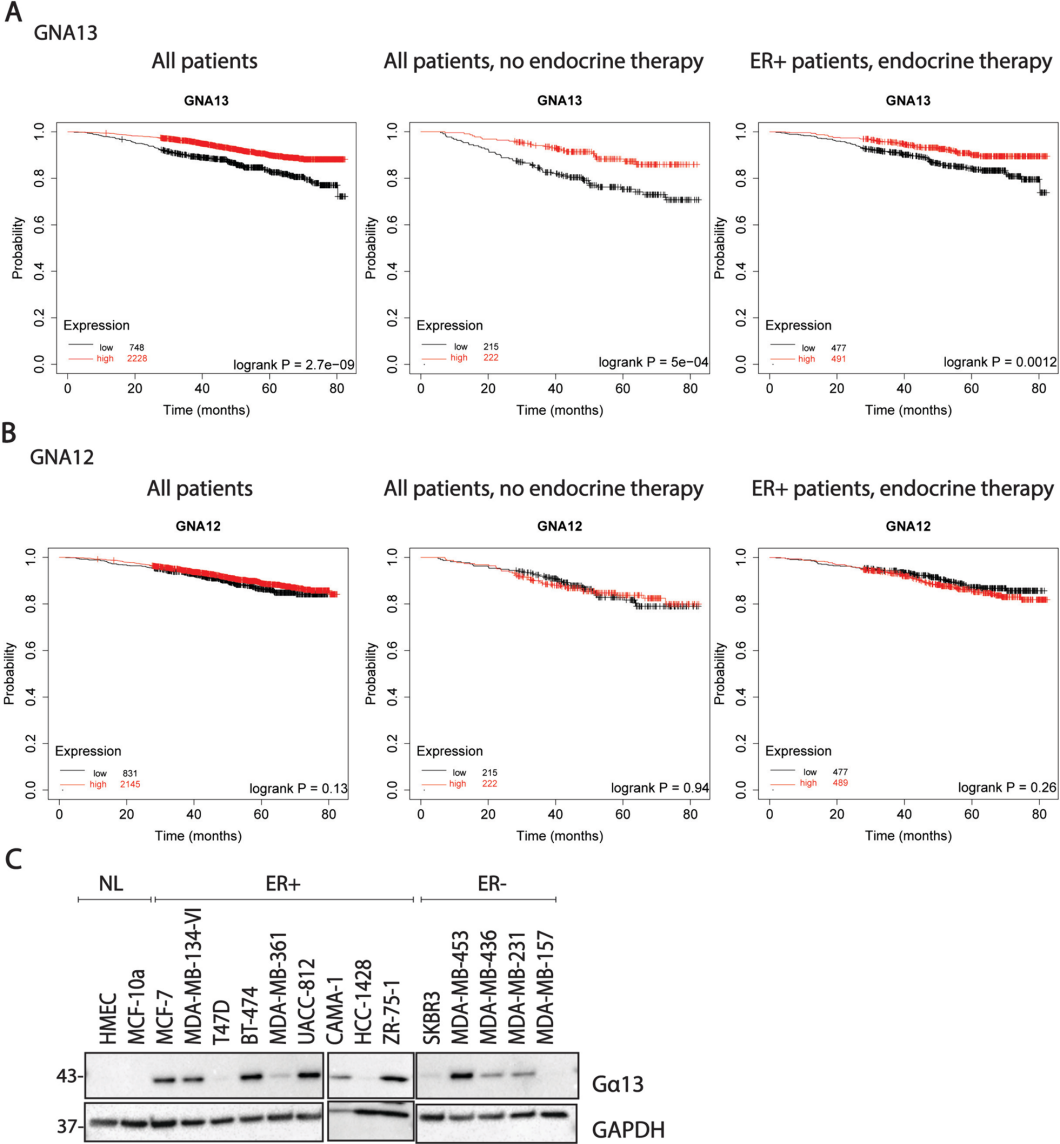
High Gα13 expression predicts better survival in breast cancers. **A** Kaplan–Meier plot showing the association between expression of *GNA13* and overall survival in breast cancer in patients in response to all treatment modalities (left); treatments other than endocrine therapy (center) or ER+ breast cancer patients given endocrine therapy (right) **B** Kaplan–Meier plot showing the association between expression of *GNA12* and overall survival in breast cancer in patients in response to all treatment modalities (left); treatments other than endocrine therapy (center) or ER+ breast cancer patients endocrine therapy (right) (data obtained from Kmplotter, www.kmplot.com) **C** Immunoblot showing the levels of Gα13 in a panel of breast cancer cell lines. HMeC, MCF-10a are non-tumorigenic. MCF-7, T47D, MDA-MB-134-VI belong to luminal A subtype (ER +). BT-474, MDA-MB-361, UACC-812 are luminal B(ER+), SKBR3 and MDA-MB-453 are ER- Her2+ and MDA-MB-436, MDA-MB-231 and MDA-MB-157 belong to TNBC. These immunoblots are representative of three independent experiments

We first examined the correlation between *GNA13* expression (TCGA) and overall survival in breast cancer using KMplotter [33, 34]. Interestingly, high *GNA13* expression significantly correlated with better survival rates in all breast cancer patients regardless of treatment group, in patients who underwent treatments other than endocrine therapy, in ER+ patients who underwent endocrine therapy (Fig. 1A) and also in ER- patients (Fig. S1A). In contrast, *GNA12* levels did not correlate with patient survival in any of the above groups (Fig. 1B). We then surveyed a small panel of ER+ and ER− cells and found that Gα13 levels varied widely among cell lines regardless of the ER status (Fig. 1C). Based on the interesting notion that higher *GNA13* expression predicts better survival, different from the previous expectation and from its family member *GNA12*, we focused on the characterization of the role of Gα13 in breast cancer cells using selected Gα13-high and Gα13-low cell line models.

### Gα13 negatively regulates proliferation of ER+ breast cancer cells

**Fig. 2:**
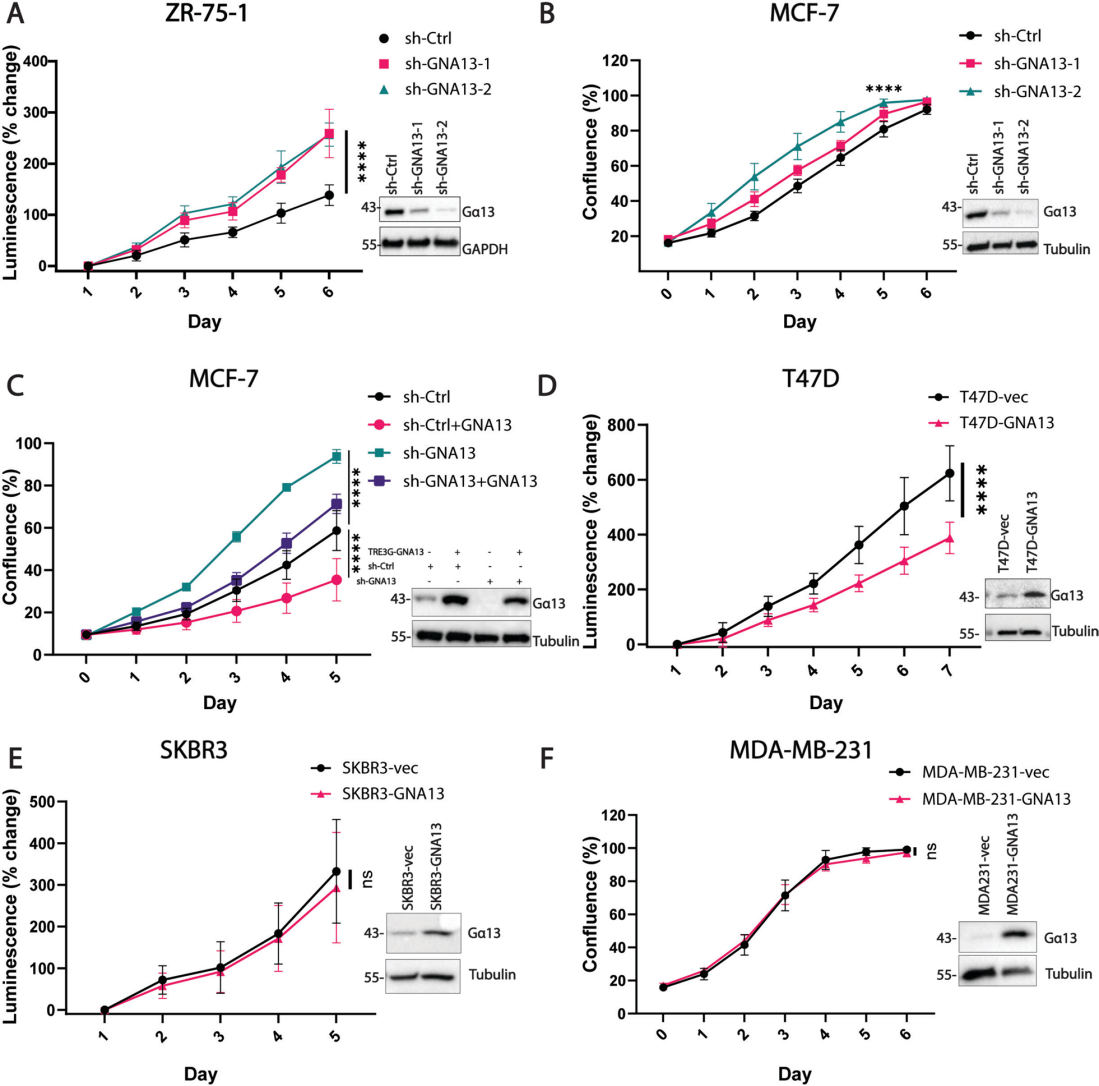
Gα13 negatively impacts proliferation uniquely in ER+ breast cancer cells. **A** Viability assay to measure proliferation of ER+ ZR-75-1 cells expressing control shRNA or that targeting *GNA13* as indicated. (Inset) immunoblot showing levels of Gα13 in the respective ZR-75-1 cell lines. **B** Proliferation of ER+ MCF-7 cells, expressing control shRNA or that targeting *GNA13* as indicated, determined by confluence measurements using the live cell imaging platform IncuCyte®. (Inset) Immunoblot showing levels of Gα13 in the respective MCF-7 cell lines. **C** Proliferation of MCF-7 knockdown cells upon reintroduction of *GNA13* as indicated, determined as in (**B**) (Inset) Immunoblot showing expression of Gα13 in the respective MCF-7 cell lines. **D** Cell viability assay to measure proliferation of ER+ T47D cells, expressing vector alone or that harboring *GNA13*, as indicated. (Inset) Immunoblot showing levels of Gα13 in the indicated ER+ T47D cell lines. **E** Proliferation of ER-/Her2+ SKBR3 cells expressing vector only or that containing *GNA13*. (Inset) Immunoblot showing levels of Gα13 in the respective SKBR3 cell lines. **F** Proliferation of ER−/Her2− MDA-MB-231 cells expressing either vector or that harboring *GNA13*. (Inset) Immunoblot showing levels of Gα13 in the respective MDA-MB-231 cells. All results shown are pooled data from three independent experiments. Data is presented as mean ± SD, and *p*-values are denoted as: *, *p* < 0.05, **, *p* < 0.01, ***, *p* < 0.001, and ****, *p* < 0.0001 or ‘ns’ for ‘not significant’. All Immunoblots are representative images of three independent experiments of the cells from the corresponding proliferation assays. See Experimental Procedures for details

We next investigated the function of Gα13 on the proliferation of ER+ T47D, MCF7 and ZR-75-1 and ER− SKBR3 and MDA-MB-231 breast cancer cells. For *GNA13*-high MCF-7 and ZR-75-1 cells, stable *GNA13* knockdown was established using retroviral transduction of short hairpin RNAs. For *GNA13*-low T47D, SKBR3 and MD-MB-231 cells, stable physiological level of expression of *GNA13* was established using a lentiviral vector with human *GNA13* coding sequence. In the ER+ *GNA13*-high ZR-75-1 and MCF-7 cells, knockdown of *GNA13* resulted in a significant increase in cell proliferation (Fig. 2A and B). To further validate that the effect of knockdown on cell proliferation is target specific, we established an inducible *GNA13* expression model in MCF-7 cells with stable *GNA13* knockdown. Exogenous expression of *GNA13* in MCF-7-shCtrl cells resulted in lowered cell proliferation, and reintroduction of *GNA13* in MCF-7-sh-*GNA13* cells resulted in a reversal of elevated cell proliferation resulting from *GNA13* knockdown (Fig. 2C). This rescue experiment provided support for the target-specific role of Gα13 in suppressing cell proliferation. Consistently, exogenous expression of *GNA13* in the *GNA13*-low ER+ T47D cells led to a marked decrease in cell proliferation (Fig. 2D). These proliferation results are in line with database analysis that indicates high *GNA13* expression predicts better overall survival in breast cancers (Fig. 1A), but in contrast to the past understanding that Gα13 is oncogenic in solid tumors and has a pro-proliferative effect. This novel notion underscores the importance of evaluating Gα13 function in different cancers, even in different subtypes of cancers.

We then expanded the study to include two ER− *GNA13*-low cell lines—SKBR3 and MDA-MB-231 cells. Comparing control and *GNA13* stable expressing cells, we found that, in contrast to the ER+ cells as shown above, exogenous expression of *GNA13* in these ER- cells had no impact on cell proliferation (Fig. 2E and F), suggesting that the effect of Gα13 on cell proliferation could be specific to ER+ breast cancer subtype. As ER+ type accounts for the majority of breast cancers and the role of Gα13 in this group is understudied, we focused on mechanistically elucidating its tumor suppressive role in the ER+ subtype for the reminder of the study.

### Gα13 negatively regulates soft agar colony formation and in vivo tumorigenesis of ER+ breast cancer cells

**Fig. 3:**
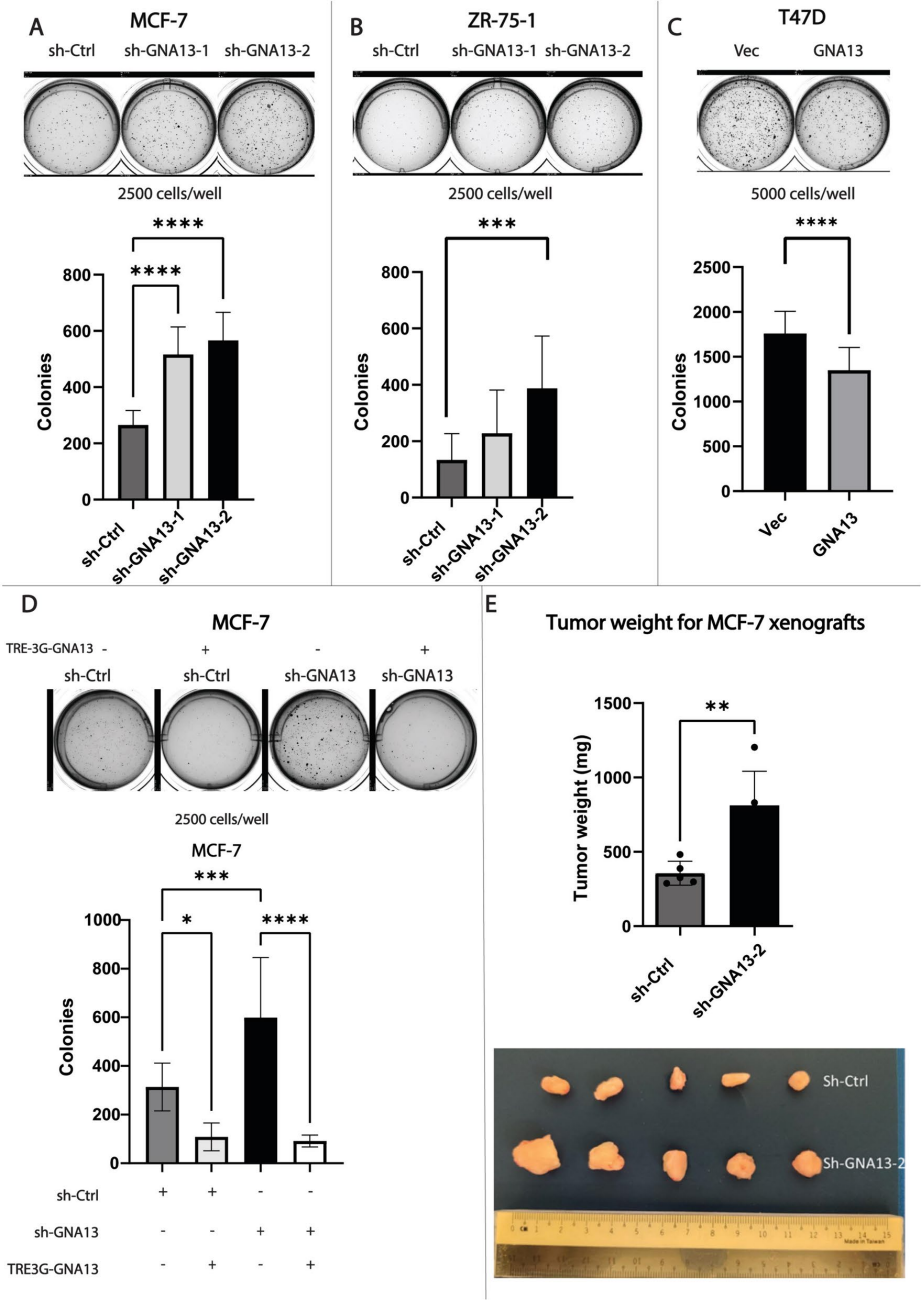
Gα13 negatively impacts soft agar colony formation and in vivo tumorigenesis in ER+ breast cancer cells. **A** Soft agar colony formation in MCF-7 cells. MCF-7 cells, expressing control shRNA or that targeting *GNA13* as indicated, were subject to soft colony formation assay as described in Experimental Procedures. Top: image showing colonies formed 21 days post seeding. Bottom: quantification of the number of colonies formed. **B** Soft agar colony formation in ZR-75-1 cells. ZR-75-1 cells expressing control shRNA or that targeting *GNA13* as indicated, were subject to soft agar colony as in (**A**). Top: image showing colonies formed 21 days post seeding. Bottom: quantification of the number of colonies formed. **C** Soft agar colony formation in T47D cells. T47D cells, expressing vector alone or that harboring *GNA13* as indicated, were subject to soft agar colony as in (**A**). Top: image showing colonies formed 21 days post seeding. Bottom: quantification of the number of colonies formed. **D** Soft agar colony formation assay in MCF-7 knockdown cells following reintroduction of *GNA13*. MCF-7 GNA13 knockdown cells, expressing vector alone or that harboring *GNA13* as indicated, were subject to soft agar colony as in (**A**). Top: image showing colonies formed 21 days post seeding. Bottom: Quantification of the number of colonies formed. **E** Top: quantification of weight of tumors at the endpoint of in vivo tumor formation studies. Bottom: images of tumors post excision. For **A**–**D**, results shown are pooled data from three independent experiments. Plotted data is presented as mean ± SD, and *p*-values are denoted as: *, *p* < 0.05, **, *p* < 0.01, ***, *p* < 0.001, and ****, *p* < 0.0001 or ‘ns’ for ‘not significant’. All colony images are representative of three independent experiments. See Experimental Procedures for details

Anchorage independent growth is a characteristic feature of tumorigenic cells; hence it is widely used as an in vitro assay to assess tumorigenicity of cancer cells. Consistent with the adherent cell culture proliferation results, knockdown of *GNA13* resulted in increased colony formation in the *GNA13*-high MCF-7 (Fig. 3A) and ZR-75-1 cells (Fig. 3B), whereas overexpression of *GNA13* in GNA13-low T47D cells resulted in decreased colony formation (Fig. 3C). Also, in line with the proliferation data, reintroduction of *GNA13* in MCF-7-sh-GNA13 cells resulted in a reversal of the increased colony formation resulting from *GNA13* knockdown (Fig. 3D). We then carried the assessment forward to in vivo tumor formation in an orthotopic xenograft mouse model. Consistent with the in vitro observations, loss of *GNA13* expression in MCF-7 cells resulted in significantly larger tumors when injected into mammary fat pad of female mice (Fig. 3E). Taken together, these results provide further evidence to support Gα13 as a critical regulator of cell growth in ER+ cells, and that expression of GNA13 alone is sufficient to suppress proliferation in this group of breast cancer cells.

### GNA13 suppresses MYC signaling in ER+ breast cancer cells

**Fig. 4:**
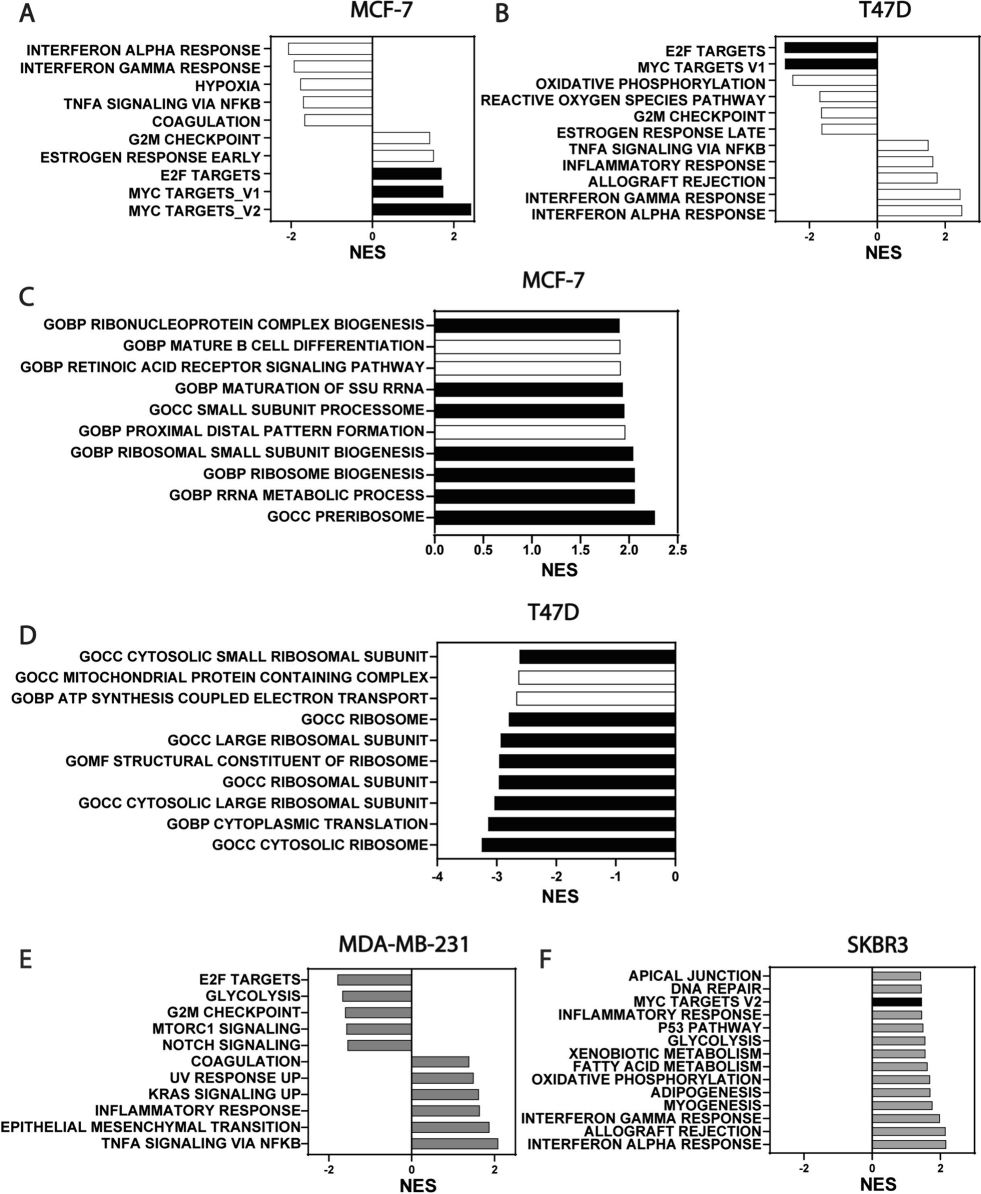
RNA sequencing analysis of ER+ breast cancer cells reveal a connection between GNA13 expression and Myc-related signaling pathways. For all experiments, cells were harvested at 80% confluence and processed as described in Experimental Procedures. **A** RNA sequencing analysis of MCF-7 sh-Control cells and those in which *GNA13* was silenced with sh-GNA13-2. Shown are the results of GSEA Hallmark analysis showing the top five pathways up-and downregulated upon GNA13 silencing. **B** RNA sequencing analysis of T47D expressing either vector or that harboring*-GNA13*. Shown are the results of GSEA Hallmark analysis showing top five pathways up-and downregulated upon *GNA13* overexpression in T47D cells. **C** Results of GSEA GO analysis showing top pathways upregulated upon *GNA13* knockdown in MCF-7 cells from (**A**). **D** Results of GSEA GO analysis showing top pathways downregulated upon *GNA13* overexpression in T47D cells from (**B**). **E** RNA sequencing analysis of MDA-MB-231 expressing either vector or that harboring *GNA13*. Shown are the results of GSEA Hallmark analysis showing the top five pathways up-and downregulated upon GNA13 overexpression in MDA-MB-231 cells. **F** RNA sequencing analysis of SKBR3 expressing either vector or that harboring *GNA13*. Shown are the results of GSEA Hallmark analysis showing top pathways upregulated upon *GNA13* overexpression in SKBR3 cells. Pathways highlighted in black represent MYC and related pathways. All pathways represented have nominal *p*-value < 0.05 and FDR < 0.25. All RNA sequencing experiments were performed in triplicate

To further investigate the mechanism of Gα13 involvement in the regulation of proliferation in ER+ breast cancer cells, we performed RNA-sequencing analysis on *GNA13*-high MCF-7 cells harboring either control shRNA or shRNA against *GNA13*, as well as on *GNA13*-low ER+ T47D, and ER- MDA-MB-231 and SKBR3 cells stably expressing either vector or *GNA13*. In the MCF-7 cells, geneset enrichment analysis using the GSEA platform (https://www.gsea-msigdb.org/gsea/msigdb/human/collections.jsp) revealed an upregulation of MYC signaling upon *GNA13* knockdown (Fig. 4A, S2A). The E2F pathway, which is well known to be related to MYC signaling, was also elevated upon *GNA13* knockdown (Fig. 4A). In agreement with the results in MCF-7, analysis of T47D cells revealed a suppression of MYC and E2F signaling pathways upon overexpression of *GNA13* (Fig. 4B, S2B).

Further enrichment analysis of the RNA-seq data using Gene Ontology was carried out to identify the biological processes and cellular components impacted by manipulation of *GNA13* expression in these ER+ breast cancer cells. This analysis indicated that changes in *GNA13* levels had significant impact on several Ribosome related pathways including RIBOSOME, RIBOSOME_BIOGENESIS and RRNA_PROCESSING (Fig. 4C and D). These findings increase the confidence on the impact of Gα13 on MYC signaling as MYC is one of the predominant drivers of the ribosome biogenesis program, which has been used as a common read-out as a major downstream consequence of alteration of MYC signaling. Reinforcing the notion that the impact of GNA13 is more significant on ER+ cells, GSEA analysis on ER- MDA-MB-231 and SKBR3 cells showed no consistent MYC signature upon *GNA13* expression (Fig. 4E). The analysis on MDA-MB-231 cells, however, indicated that *GNA13* may be a driver for inflammatory response and Epithelial Mesenchymal Transition (Fig. 4E), in line with our previous findings that suggest Gα13 involvement in NFκB signaling in TNBC [22] and prostate cancer cells [21]. The analysis of SKBR3 similarly suggested that *GNA13* overexpression mainly affects inflammatory response pathways, with minor effect on MYC signaling (Fig. 4F), consistent with the notion that Gα13 exerts proliferative effect mainly on ER+ cells. In summary, the comparative analysis of top altered pathways upon manipulation of *GNA13* expression in four different breast cancer cell lines (MCF-7, T47D, MDA-MB-231 and SKBR3) indicated that Gα13 regulation of MYC signaling, particularly ribosomal biogenesis is unique to ER+ cell (Fig. S2C, highlighted in red).

### Gα13 regulates MYC expression in ER+ breast cancer cells

**Fig. 5:**
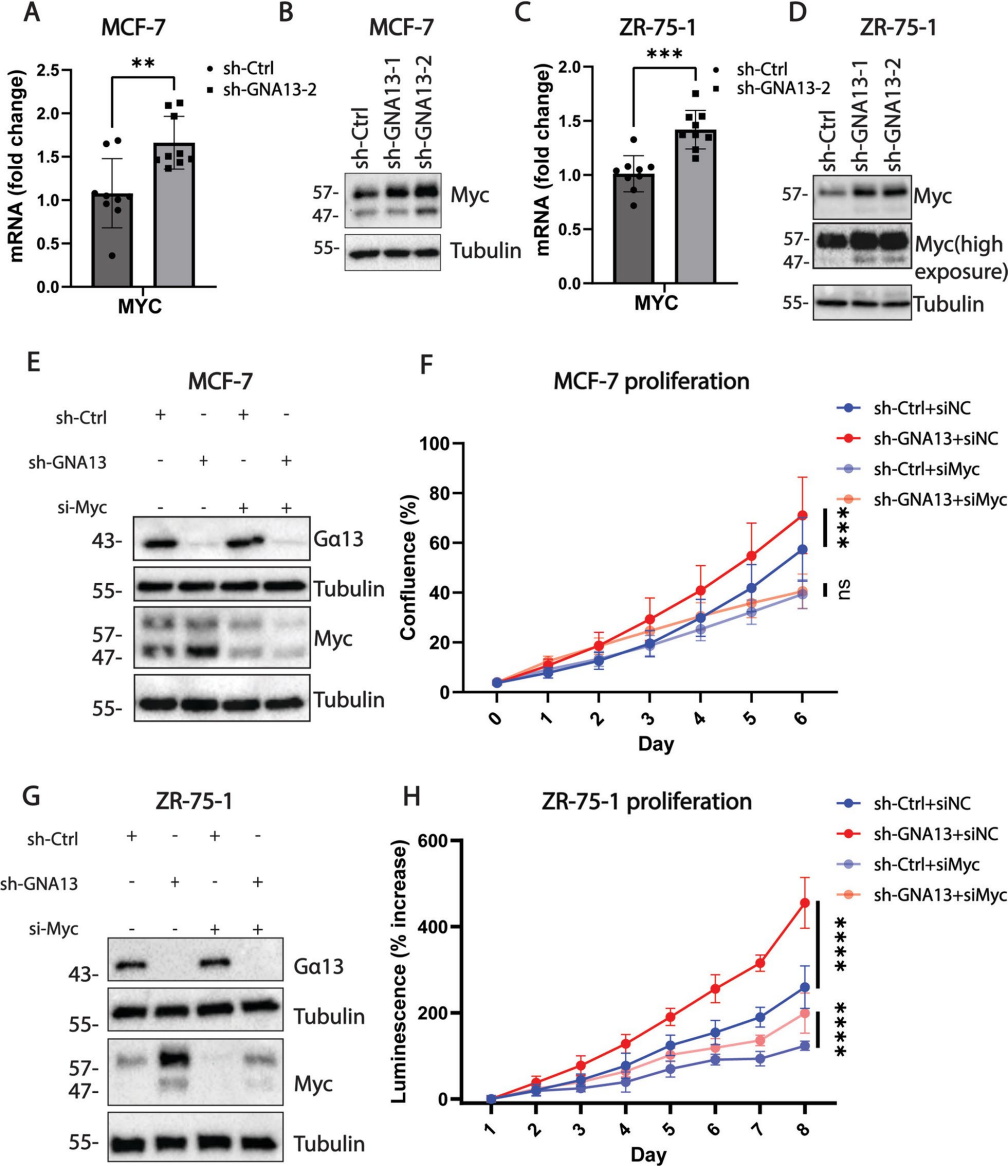
Gα13 suppresses the expression of MYC, and loss of MYC reverses the proliferative phenotype observed upon GNA13 silencing in ER+ breast cancer cells. **A** *MYC* mRNA levels in MCF-7 cells upon *GNA13* silencing, RNA levels were assessed by real-time PCR; relative mRNA expression plotted as fold-change to control cells (sh-GNA13-2 compared to sh-control), *HPRT* was used a normalizing control. **B** Immunoblot showing the expression of MYC upon *GNA13* silencing in MCF-7 cells. **C** *MYC* mRNA levels in ZR-75-1 cells upon *GNA13* silencing, RNA levels were assessed as in (**A**). **D** Immunoblot showing the expression of MYC upon *GNA13* knockdown in ZR-75-1 cells. **E** Immunoblot showing Gα13 and MYC levels in MCF-7 cells (sh-Control and sh-GNA13-2) with or without silencing of *MYC*. **F** proliferation of ER+ MCF-7 cells in (**E**) as determined by confluence measurements using the live cell imaging platform IncuCyte®. **G** Immunoblot showing Gα13 and MYC levels in ZR-75-1 cells (sh-Control and sh-GNA13-2) with or without silencing of *MYC*. **H** Proliferation of ER+ ZR-75-1 cells in (**G**). Results shown are pooled data from three independent experiments. Plotted data is presented as mean ± SD, and *p*-values are denoted as: *, *p* < 0.05, **, *p* < 0.01, ***, *p* < 0.001, and ****, *p* < 0.0001 or ‘ns’ for ‘not significant’. All immunoblots are representative of three independent experiments. See Experimental Procedures for details

After the identification of MYC associated pathway signature from the RNA-seq data, we evaluated the expression of the *MYC* oncogene and found that its expression is significantly increased upon *GNA13* silencing in MCF-7 cells (Fig. S3A). Validation study confirmed that both the transcript levels (Fig. 5A and C) and protein levels (Fig. 5B and D) are elevated upon *GNA13* knockdown in *GNA13*-high—MCF-7 and ZR-75-1 cells, consistent with the findings from RNA-sequencing analysis. In addition, we found that the changes in the Myc levels also translates into the changes in Myc activity, as consistent changes are observed in Myc downstream pathways and genes (Supplementary Fig. S3B, C). In *GNA13*-low T47D cells, we also found that overexpression of *GNA13* results in the selective suppression of the smaller MYC isoform (Fig. S3D), which, of the two isoforms has been shown to be predominantly responsible for oncogenic and proliferative properties of the MYC oncogene [35, 36].

To investigate whether the increase in MYC mediates the effect on proliferation upon *GNA13* knockdown, we assessed the effect of concurrent silencing of *MYC* and GNA13 on cell proliferation in *GNA13*-high MCF-7 and ZR-75-1 cells. As shown earlier, *GNA13* knockdown alone led to elevated MYC protein levels (Fig. 5E and G) and increased proliferation (Fig. 5F and H). Concurrent *MYC* knockdown significantly reversed the increase in proliferation brought on by silencing of *GNA13* in both MCF-7 (Fig. 5F) and ZR-75-1 (Fig. 5H) cells. Together, these results demonstrate that Gα13 controls MYC-regulated processes by modulating *MYC* expression, through which Gα13 plays a significant role in proliferation.

### Gα13 regulation of MYC expression is ERα dependent, which accounts for this ER+ specific regulation of proliferation

So far, both RNA-seq and phenotypic assays on multiple cell lines suggest that the Gα13-MYC signaling mechanism is specific to ER+ breast cancer cells, which raises the possibility of the involvement of estrogen and/or estrogen receptor-dependent regulation in this novel Gα13-MYC signaling axis. As a major oncogene, *MYC* expression is regulated at transcriptional, post transcriptional and translational levels across all subtypes of breast cancers [37]. In ER+ cells, *MYC* expression is reported to be predominantly driven by estrogen signaling [38, 39] and the *MYC* gene is a direct transcriptional target of ESR1 (ERα) [40]. In this regard, additional analysis of our RNA-sequencing data from MCF-7 consistently revealed the upregulation of several estrogen signaling related pathways upon *GNA13* silencing (Fig S4A, B) and *MYC* was one of the most significantly upregulated estrogen response targets in MCF-7 cells impacted by *GNA13* (Fig. S4C). Further, transcription factor analysis of the genes contributing to the estrogen signature revealed a significant overlap with known *MYC* targets (Fig S4D). Based on the data, the possibility is raised on whether Gα13 regulation of MYC is mediated by ESR1, which would fit with the Gα13 effect on proliferation being limited to ER+ cells.

**Fig. 6:**
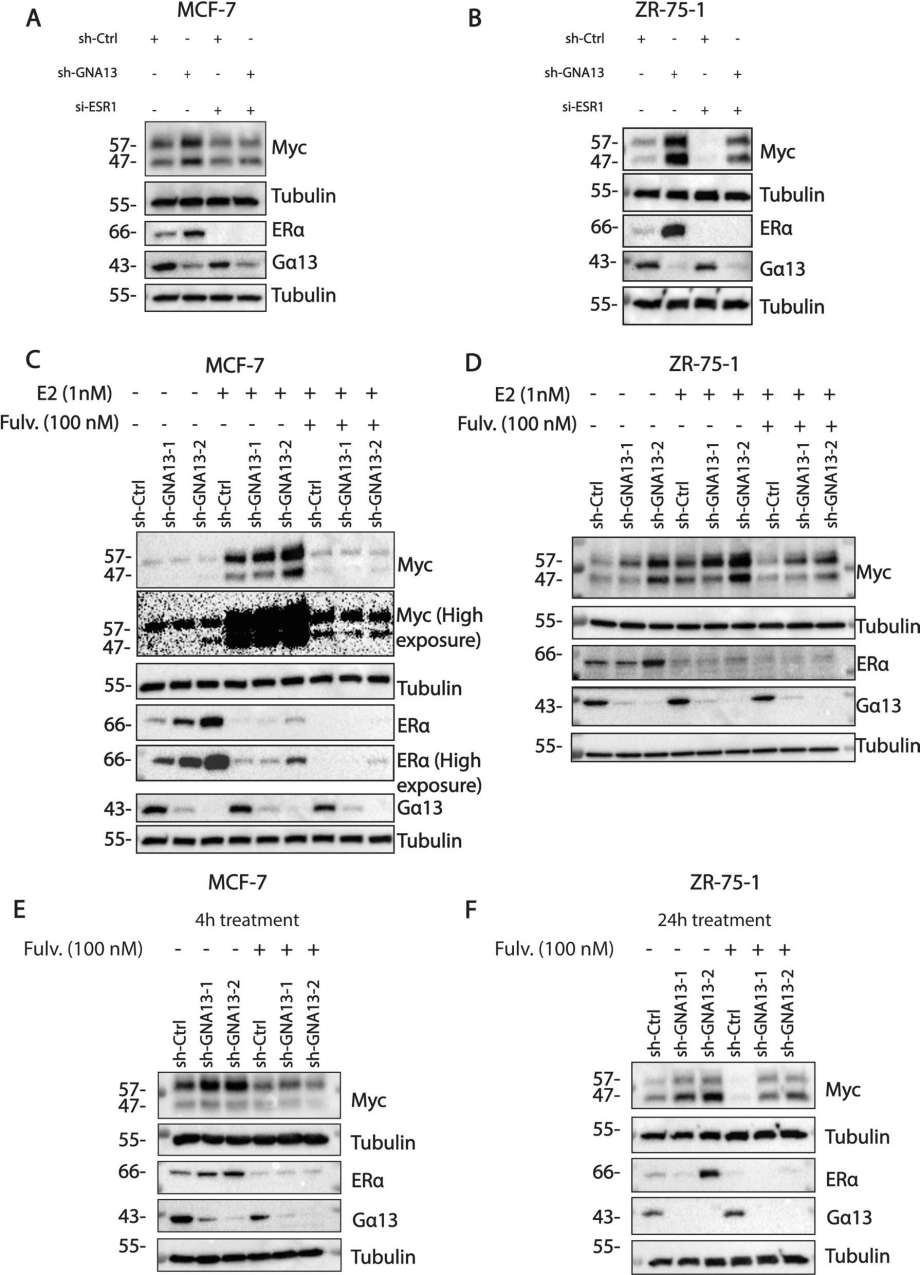
Increased levels of *MYC* observed upon GNA13 loss is context dependent. **A** Immunoblot showing the levels of MYC upon *ESR1* silencing in MCF-7 sh-Control and sh-GNA13 cells. **B** Immunoblot showing the levels of MYC upon *ESR1* silencing in ZR-75-1 sh-Control and sh-GNA13 cells. **C** Immunoblot showing estradiol dependent induction of MYC in MCF-7 cells, MCF-7 cells were deprived of estradiol by treating in Charcoal-Stripped FBS media for 72 h, and then stimulated with either 1 nM E2 in presence or absence of 100 nM fulvestrant for 6h. **D** Immunoblot showing estradiol dependent induction of MYC expression in ZR-75-1 cells, ZR-75-1 cells were deprived of Estradiol by treating in Charcoal-Stripped FBS media for 48 h, and then stimulated with either 1 nM E2 in presence or absence of 100 nM fulvestrant for 6h. **E** Immunoblot showing the levels of MYC in MCF-7 cells (sh-Control, sh-GNA13-1, shGNA13-2) cells upon estrogen signaling inhibition using 100 nM Fulvestrant for 4h. **F** Immunoblot showing the levels of MYC in ZR-75-1 cells (sh-Control, sh-GNA13-1, sh-GNA13-2) cells upon estrogen signaling inhibition using 100 nM Fulvestrant for 24h.For immunoblots, results shown are representative of three independent experiments

We then directly assessed the impact of *GNA13* knockdown on *ESR1* (ERα) expression and the role of ERα in MYC expression observed upon *GNA13* loss in *GNA13*-high MCF7 cells. As described above, knockdown of *GNA13* led to an increased expression of MYC in both cell lines. Interestingly, concurrent silencing of *ESR1* expression was sufficient to abrogate the increase in *MYC* expression observed from GNA13 knockdown (Fig. 6A). A similar phenomenon was observed in ZR-75-1 cells (Fig. 6B). This reversal of MYC expression by suppressing *ESR1* provides strong evidence for the ERα-dependence in the regulation of MYC by Gα13.

To delineate between ligand dependent and independent ERα signaling in these ER+ cells, we also studied the regulation of *MYC* by Gα13 in sterol deprived conditions, followed by addition of estradiol (E2). We found that, in the absence of E2, both ERα and MYC expression are induced when *GNA13* is silenced, albeit the induction of MYC is at a more subdued level (Fig. 6C). The expression of ERα increased at comparable level as in the normal growth medium (Fig. 6C compared to Fig. 6A), which suggests that Gα13 regulation of ERα expression is largely ligand independent (Fig. 6C). Under E2 treatment, the expression of *MYC* was significantly greater than in sterol deprived media and appears to be at comparable level to that in the normal medium (Fig. 6C compared to Fig. 6A), suggesting a critical role of upstream estrogen signaling in regulating MYC in these cells. Noteworthy, loss-of-GNA13 still had significant effect on MYC expression. Further evaluation with ERα antagonist fulvestrant added to the sterol-depleted medium demonstrate complete loss of MYC induction upon *GNA13* knockdown suggesting a dependency on ligand stimulated ERα in this regulation; and it appears likely that GNA13 and ERα work together to regulate MYC in these cells. Under estradiol stimulation, the levels of ERα as well as its induction on *GNA13* knockdown are more subdued than that in the absence of E2 (Fig. 6C). The tapered level of ERα in the presence of E2 is consistent with the understanding that ligand binding of ERα leads to its polyubiquitination and degradation.

The above observations held true for the ER+ ZR-75–1 cells as well, in all of the important aspects as observed in MCF7 cells (Fig. 6D). First, loss of *GNA13* led to an increase in ERα and MYC in all treatment conditions; second, the presence of E2 tapers the ERα level while further inducing the MYC level. Ligand binding to activate the receptor appears to induce a more pronounced induction of MYC as compared to the slight induction in the absence of ligand. This supports the notion that this GNA13-ERα-MYC axis of regulation of proliferation is a broader feature of ER+ cells.

Our results suggest that the levels of ligand present in normal medium (containing FBS) is sufficient to significantly induce MYC expression and the ensuing proliferation in these ER+ cells (Fig. 6A and B). To further evaluate this, we also compared the expression of ERα and MYC in normal FBS containing medium with and without the addition of the antagonist fulvestrant to observe the dependency on ERα activation for the induction of MYC expression by *GNA13* knockdown. Fulvestrant reduces both the baseline and the induction of ERα expression from *GNA13* knockdown, leading to the inhibition of basal and induced MYC expression, suggesting estrogen signaling is the predominant pathway responsible of MYC expression in normal medium as well (Fig. 6E and F).

Taken together, our findings demonstrate that ERα regulates *MYC* expression, and that the impact of Gα13 on MYC expression is, for the most part, driven through the E2-ERα signaling axis. In both MCF-7 and ZR-75-1 cells, the results from the treatments under both normal growth, sterol deprived, E2 stimulated and fulvestrant inhibited conditions allow us to conclude that Gα13 regulation of MYC expression is dependent on ERα. The activation of ERα by agonist further enhances the promotion of MYC expression and the inactivation of ERα by antagonist abrogates the induction of MYC. We therefore speculate that the ER+ subtype specific impact on proliferation and induction of MYC by GNA13 lies in the ability of the ERα receptor activation by ligand present in the normal media and in the circulation in vivo.

## Discussion

Breast cancer is currently the most diagnosed cancer worldwide and continues to be one of the leading causes of cancer related deaths although outcomes have significantly improved since the advent of endocrine therapy. As over 80% of all breast cancers are hormone dependent, anti-estrogens such as tamoxifen or aromatase inhibitors such as Letrozole have been the mainstay of treatment and have been largely responsible for dramatically improving survival rates in this type of breast cancer. However, it is now accepted that resistance to endocrine therapy is an inevitable occurrence and currently it accounts for the largest proportion of breast cancer related deaths. The emergence of endocrine therapy resistance is now widely understood to be a complex process and there is an urgent need to unravel the key determinants of progression to endocrine resistant breast cancer. Most importantly, at 80% of the total breast cancer burden, better understanding of ER+ breast cancer should improve therapeutic efficacies and overall survival of breast cancer in general.

The G12 subfamily of heterotrimeric G-proteins GNA12 (Gα12) and GNA13 (Gα13) are ubiquitously expressed and are known to regulate major cell signaling pathways that regulate actin-cytoskeleton remodeling and cell proliferation processes important for cancer cells. As such, *GNA12* and *GNA13* have been shown to be overexpressed in multiple tumor types such as liver, gastric, head and neck squamous cell carcinomas among others [12, 15, 41, 42]. Nonetheless, it is becoming increasingly apparent that G12/G13 family proteins, particularly Gα13 can have context dependent roles in cancer. It has been known for some time that unlike in solid tumors, in B-cell Lymphomas (DL-BCL) Gα13 functions as a tumor suppressor [43] and more recently Gα13 has also been shown to have a tumor suppressive role in KPC mouse model of pancreatic cancer [28]. We have previously shown that Gα13 drives the invasion of triple negative breast cancer (TNBC) cells in vitro [22]. However, breast cancer is a highly heterogenous disease with widely varying vulnerabilities and outcomes in each of the subtypes, and functions of the G12 proteins remain largely unexplored outside of the TNBC subtype which constitutes a very small fraction of patients. In this study, we explored the function of Gα13 in breast cancers by analyzing the prognostic value of *GNA13* expression level in breast cancers.

In most solid tumors, increased levels of the wild type Gα13 has been observed in more aggressive cancers and is known to drive stemness, invasiveness and drug resistance, and consequently is associated with poorer survival [27]. However, in this study, contrary to expectations, we find that higher Gα13 protein level is associated with improved survival. The validation experiments in a group of breast cancer cell lines led us to conclude that Gα13 functions as tumor suppressor in ER+ breast cancers. On the other hand, in ER-negative breast cancers despite a significant positive correlation to survival, we find that Gα13 does not impact proliferation, in addition to a previously reported oncogenic role in this subtype, therefore further investigations will be necessary. These findings suggest that the role of Gα13 signaling in cancer is more complex than previously understood and is likely dependent on the interplay between Gα13 and the various signaling networks.

In this study, we discovered that *GNA13* is a negative regulator of MYC oncogene expression; suppressing GNA13 leads to increased proliferation via upregulation of MYC signaling pathways. Deregulation of *MYC* expression is a widespread event in carcinogenesis and is reported to occur in nearly 70% of all cancers [44]. In breast cancers, *MYC* overexpression has been reported at transcriptional, post transcriptional and translational level [37]. In ER+ breast cancer cells, *MYC* expression is predominantly driven by estrogen signaling [38, 39] and is a direct transcriptional target of ERα [40]. In this study we find that *GNA13* regulates the expression of MYC exclusively in ER+ breast cancer cells, and that the induction of *MYC* observed upon *GNA13* loss is dependent on the expression and activation of ERα. This is particularly relevant in ER+ breast cancers as *MYC* is a key factor responsible for driving the effects of estrogens on cell cycle progression and has been shown to be required for E2 dependent proliferation of ER+ breast cancer cells [38, 45, 46]. Whether Gα13 contribute significantly to estrogen independent ectopic overexpression of *MYC,* which has been reported to induce proliferation of MCF-7 cells [47, 48], remains to be clarified in future studies. This topic is of significant value to expand our understanding on the development of anti-estrogen resistance in ER+ breast cancers [49, 50].

**Fig. 7:**
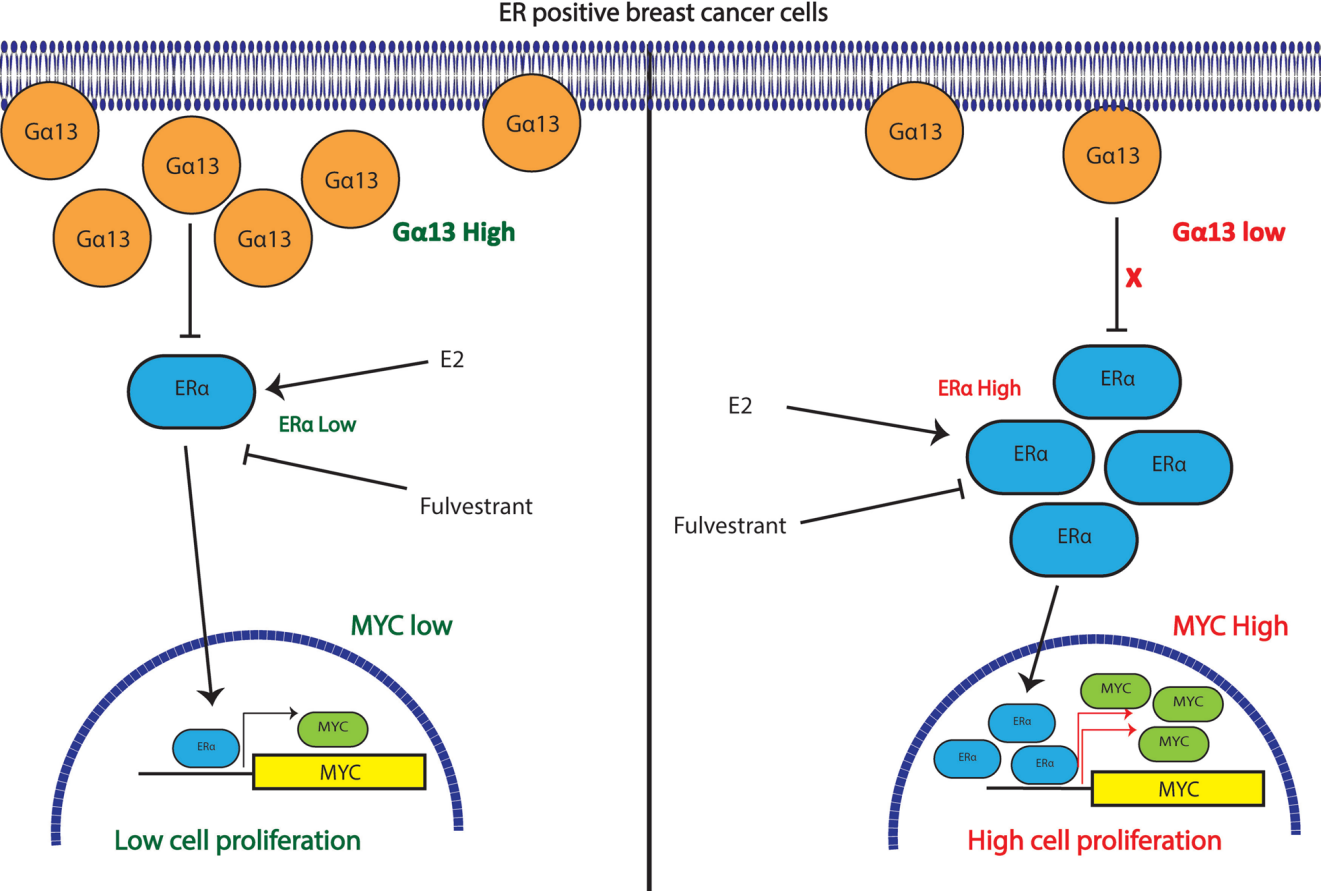
Schematic diagram showing the findings from this study: in the ER+ subtype, loss of *GNA13* results in increased proliferation and tumor formation suggesting a tumor suppressive role for *GNA13* in this subtype. This phenotype is dependent on upregulation of MYC signaling pathway observed upon *GNA13* silencing exclusively in ER+ cell lines, where loss of GNA13 drives the expression of MYC through increasing ERα driven estrogen signalling

In summary (Fig. 7), we have shown in this study that Gα13 can have subtype specific effects in breast cancer, with a focus on ER+ breast cancers. To our knowledge this is the first study to show a direct impact of Gα13 on proliferation and survival in breast cancer. In ER+ breast cancers, particularly of the Luminal A subtype, our results show that Gα13 suppresses growth of cells. Further, we show that loss of Gα13 results in an upregulation of MYC signaling pathway. Finally, we show that mechanistically, this impact on *MYC* expression driven by Gα13 is ERα dependent, uncovering a hitherto unknown Gα13-ERα-MYC signaling axis. Additionally, we also observed an increase in ERα upon GNA13 silencing in our ER+ models. We speculate that the impact of GNA13 on ERα could be driven by mechanisms involving regulation of translation possibly as a part of a feedback loop, especially since Myc is a known regulator of translation and GNA13 knockdown in our models has shown to impact several pathways involving translation. Another possibility is the involvement of post transcriptional mechanisms such as dysregulation of ERα-targeting microRNAs, which we have not ruled out. Given our findings of GNA13 regulation of the expression of several key players of ER+ breast cancer pathogenesis such as MYC and ERα that are well known to correlate to resistance to anti-estrogen treatments and emergence of Long-Term Estrogen Deprivation (LTED) characteristics, the impact of Gα13 on aspects of endocrine resistance in this subtype will be an important area for future investigations.

### Supplementary Information

Supplementary Material 1

Supplementary Material 2

## Data Availability

The datasets used and/or analyzed during the current study are available from the corresponding author(s).
