# Supplementary material for: GNA13 suppresses proliferation of ER+ breast cancer cells via ERα dependent upregulation of the MYC oncogene
Source: Breast Cancer Res. 2024 Jul 4;26:113. doi: 10.1186/s13058-024-01866-x (PMC11225210; doi:10.1186/s13058-024-01866-x)
Supplement: Supplementary file 1 — Supplementary Material 1 [file 13058_2024_1866_MOESM1_ESM.docx]

**Supplementary figure 1**


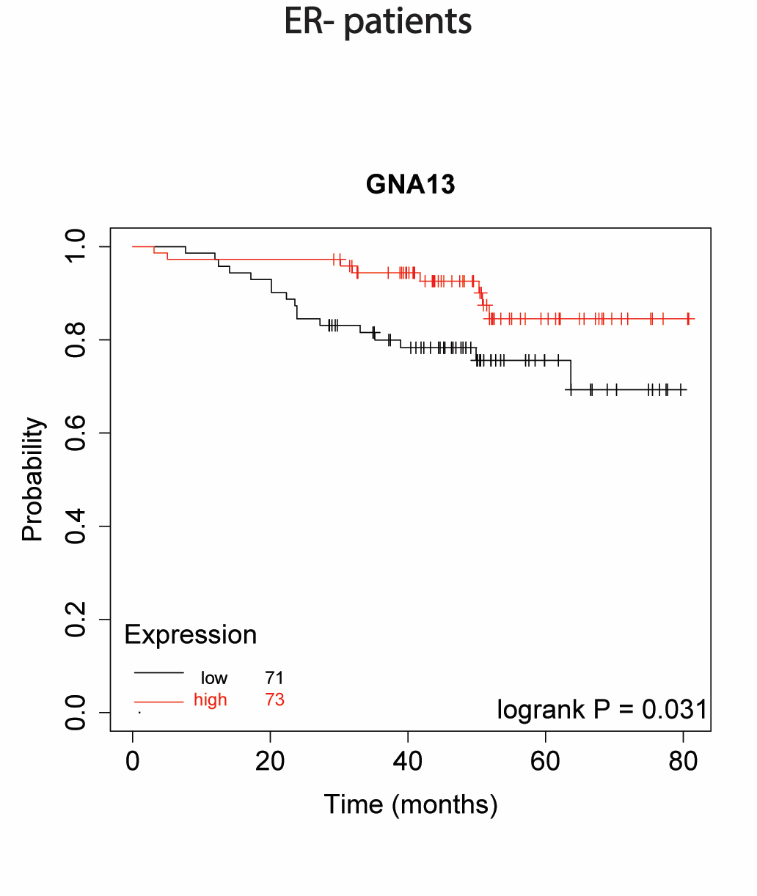


**Fig 1. High Gα13 expression predicts better survival in breast cancers. (A)** Kaplan-Meier plot showing the association between expression of *GNA13* and overall survival in breast cancer in ER- negative patients in all treatment modalities. (data obtained from Kmplotter, [www.kmplot.com](https://kmplot.com/analysis/index.php?p=service)).

**Supplementary Figure 2**


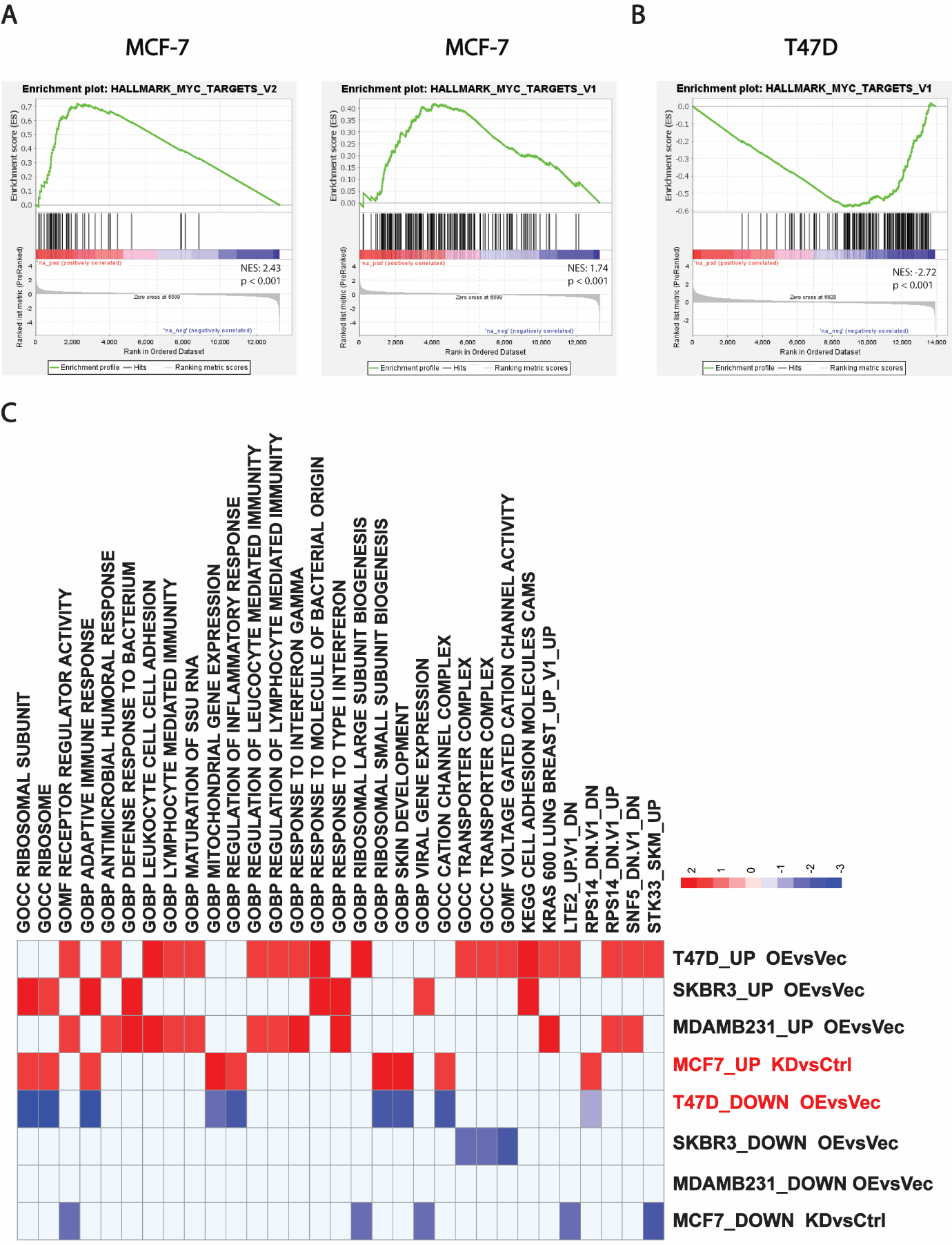


**Fig S2: GNA13 is regulates MYC signalling in ER+ breast cancers: (A)** GSEA enrichment plot showing the upregulation of genesets MYC_TARGETS_V2 (left) and MYC_TARGETS_V1 (right) upon *GNA13* silencing in MCF-7 cells. **(B)** GSEA enrichment plot showing the suppression of MYC_TARGETS_V1 upon *GNA13* overexpression in T47D cells. In A-B, the peak of the green curve indicates enrichment score of the geneset used to calculate the Normalised Enrichment Score (NES). **(C)** Heat map showing the top pathways altered upon *GNA13* silencing/overexpression across subtypes MCF-7, T47D- ER+, SKBR3-ER- Her2+, MDA-MB-231- TNBC, center two rows (labelled in red) represent the impact on Ribosome pathways observed upon altering GNA13 expression in ER+ cell lines.

**Supplementary Figure 3**


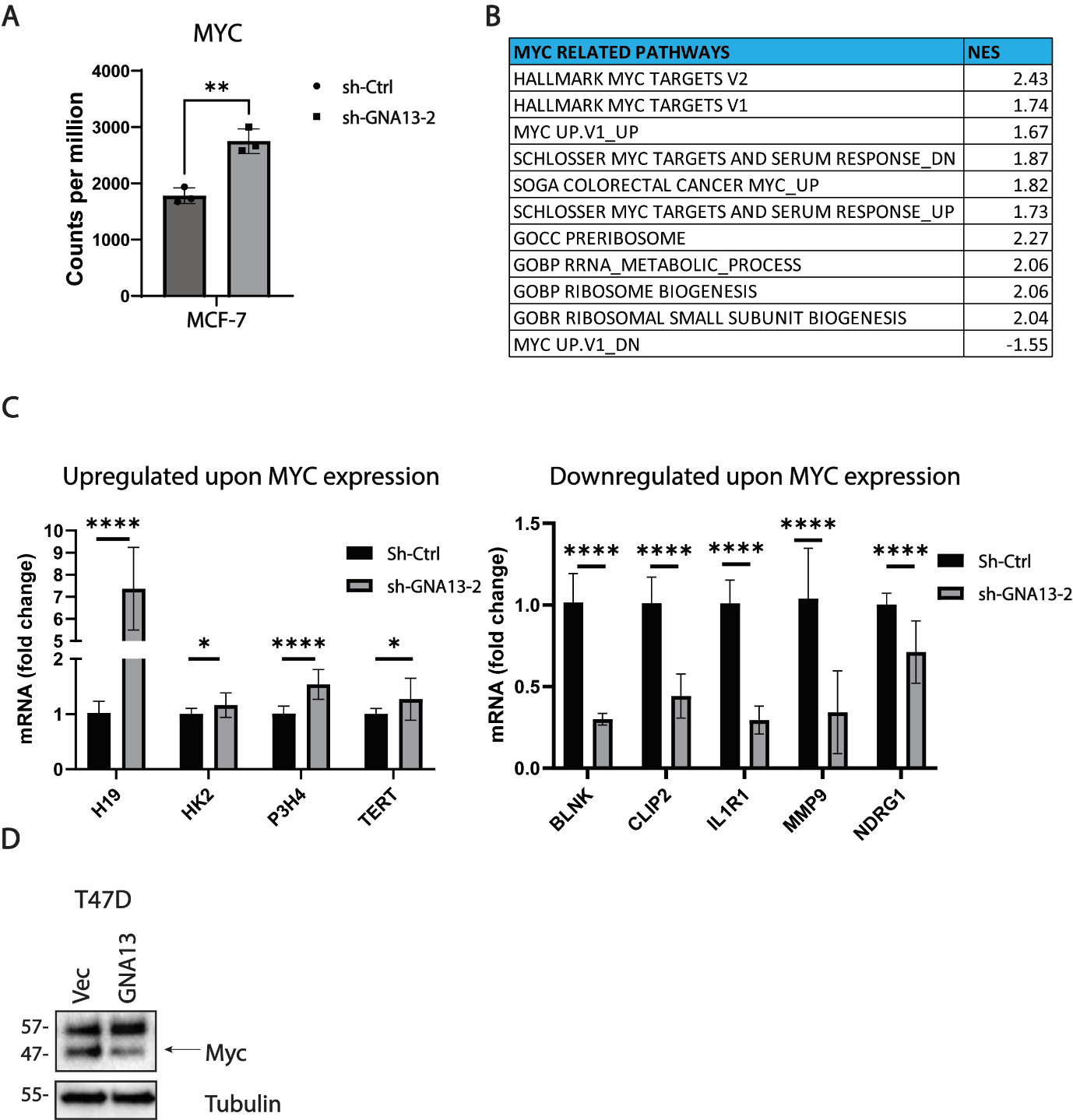


**Fig S3: GNA13 is regulates the expression of MYC in ER+ breast cancer cells: (A)** RNA expression of *MYC*, as measured by counts per million in RNA-sequencing analysis of MCF-7 cells. (**B**) Table showing MYC regulated pathways in MCF7 breast cancer cells that are significantly affected upon GNA13 knockdown. NES: normalized enrichment scores. (**C**) mRNA levels of MYC target genes in MCF-7 cells upon *GNA13* silencing, RNA levels were assessed by real-time PCR; relative mRNA expression plotted as fold-change to control cells (sh-GNA13-2 compared to sh-control), *HPRT* was used a normalizing control. **(D)** Immunoblot showing the levels of MYC in T47D cells expressing either vector or that harboring *GNA13*. Results shown are pooled data from three independent experiments. Data is presented as mean ± SD, and *p*-values are denoted as: *, *p* <0.05, **, *p* <0.01 and ***, *p* <0.001 or ‘ns’ for ‘not significant’. Immunoblot is representative image of three independent experiments.

**Supplementary Figure 4**


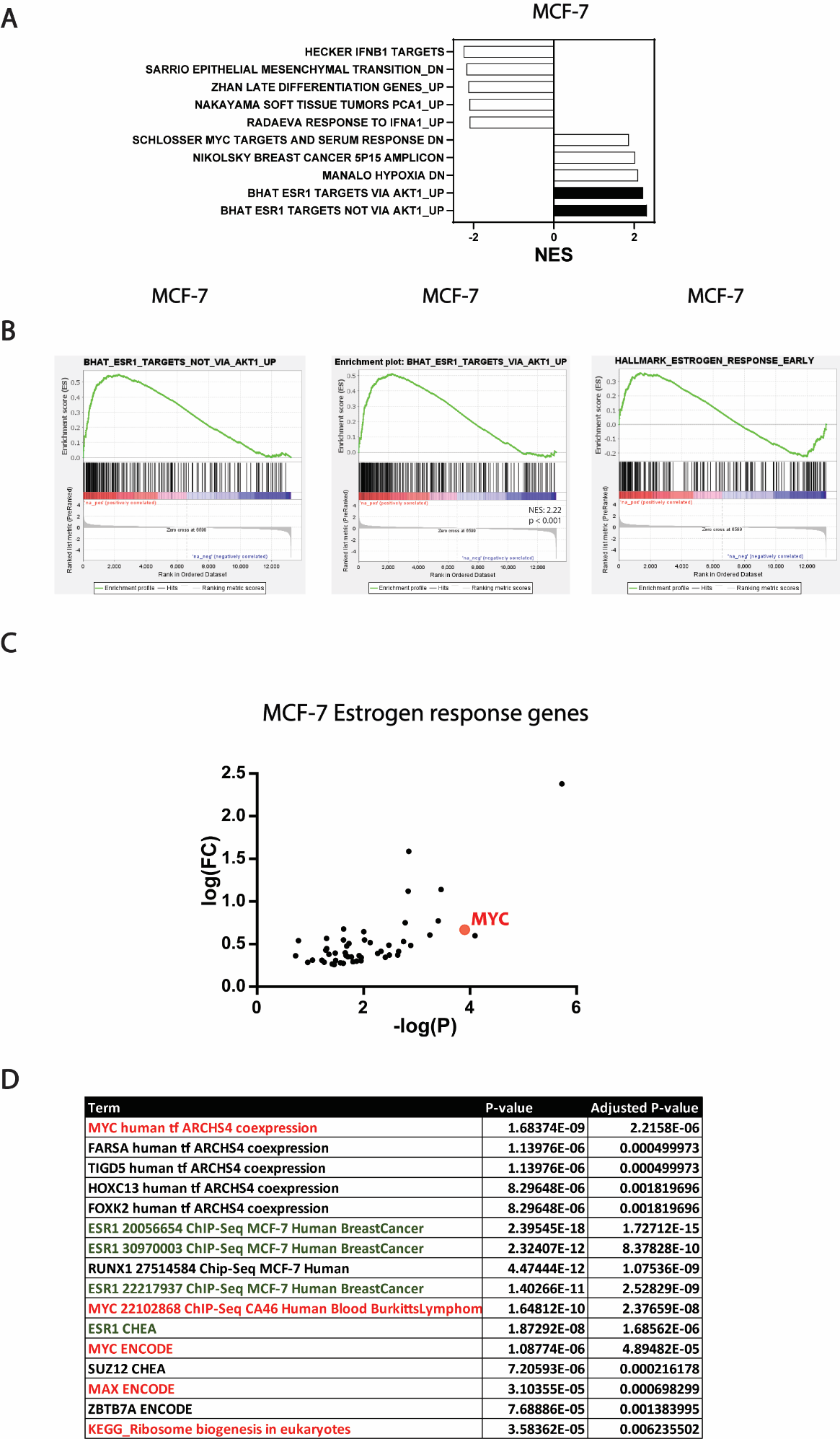


**Fig S4: Loss of GNA13 upregulates MYC-Estrogen signalling axis in MCF-7 cells:** **(A)** Results of GSEA Chemical and genetic perturbations analysis showing top five pathways up-and downregulated upon in MCF-7 cells upon *GNA13* silencing. All pathways have a nominal p-value < 0.05 and FDR < 0.25. **(B)** GSEA enrichment plot showing the upregulation of genesets from S4A and 4A related to Estrogen targets upon *GNA13* silencing in MCF-7 cells . **(C)** Scatter plot showing genes belonging to the Estrogen response genes in the GSEA Hallmarks set upregulated upon *GNA13* knockdown in MCF-7 cells. *MYC* is highlighted in red. **(D)** Table showing the transcription factor analysis of genes belonging to estrogen response genesets that are upregulated upon *GNA13* silencing, Among the top significant transcription factors are MYC and related MAX (highlighted in red) and estrogen signalling pathways (highlighted in green). Transcription factor analysis was performed using enrichr analysis tool (<https://maayanlab.cloud/Enrichr/>).

**Supplementary Table 1**

**Sequences of primers designed for cloning GNA13 in Plvx-CMV-Puro construct**

| **Primer** | **Sequence 5’ to 3’** |
| --- | --- |
| GNA13- (EcoRI) Forward | CTCAAGCTTCGAATTCATGGCGGACTTCCTGCCG |
| GNA13- (BamHI) Reverse | TAGAGTCGCGGGATCCTCACTGTAGCATAAGCTGCTTGAGG |

**Supplementary Table 2**

**Sequences of primers designed for measuring mRNA expression of various genes by real-time PCR.** HPRT is used as a normalizing control. All primers were designed to target human genes.

| **Primer** | **Sequence 5’ to 3’** |
| --- | --- |
| HPRT | Forward: CATTATGCTGAGGATTTGGAAAGG  Reverse: CTTGAGCACACAGAGGGCTACA |
| GNA13 | Quantitect® Primer Assay, QT0007968, purchased from Qiagen |
| GNA13 | Forward: TCCACCTTCCTGAAGCAGATGC  Reverse: GCTTCTCTCGAGCATCAACCAG |
| MYC | Forward: CCTGGTGCTCCATGAGGAGAC  Reverse: CAGACTCTGACCTTTTGCCAGG |

**Supplementary Table 3**

**Supplementary Table 3: Commercial si-RNAs used for transient knock-down of target genes.**

| **siRNA** | **Specifications** |
| --- | --- |
| MYC | ON-TARGETplus Human MYC (4609) siRNA -SMARTpool, Dharmacon L-003282-02-0010) |
| ESR1 | ON-TARGETplus Human ESR1 (2099) siRNA- SMARTpool, Dharmacon L-003401-00-0010 |
| Negative control | Qiagen, All stars negative control siRNA 1027281 |
| Negative control | Mission siRNA Fluorescent Universal negative control #2, 6-FAM, SIC008 |

**Supplementary Table 4**

**Antibodies used for immunoblotting.** Tubulin and GAPDH are both used as loading controls.

| **Antibody** | **Specifications** | **Species** | **Dilution** |
| --- | --- | --- | --- |
| Gα13 | Cat # ST1629, Clone 6F6-B5, purchased from Calbiochem | Mouse | 1:1000 |
| Tubulin | Cat# T5168, purchased from Sigma-Aldrich | Mouse | 1:3000 |
| GAPDH | Cat# Ab8245, purchased from Abcam | Mouse | 1:2000 |
| ERα | Cat# 8644, purchased from cell signalling technology | Rabbit | 1:1000 |
| MYC | Cat# Ab32072, purchased from Abcam | Rabbit | 1:2000 |
| Goat Anti-Mouse light chain Antibody, HRP conjugate | Cat # AP200P, purchased from Merck Millipore | Secondary antibody against Mouse | 1:3000 |
| Goat Anti-Rabbit IgG Antibody, Peroxidase Conjugated | Cat # AP132P, purchased from Merck Millipore | Secondary antibody against Rabbit | 1:3000 |
