# Supplementary material for: GNA13 suppresses proliferation of ER+ breast cancer cells via ERα dependent upregulation of the MYC oncogene
Source: Breast Cancer Res. 2024 Jul 4;26:113. doi: 10.1186/s13058-024-01866-x (PMC11225210; doi:10.1186/s13058-024-01866-x)
Supplement: Supplementary file 2 — Supplementary Material 2 [file 13058_2024_1866_MOESM2_ESM.pdf]

Uncropped western blots

Fig 1C

= Relevant samples enclosed within red for all blots

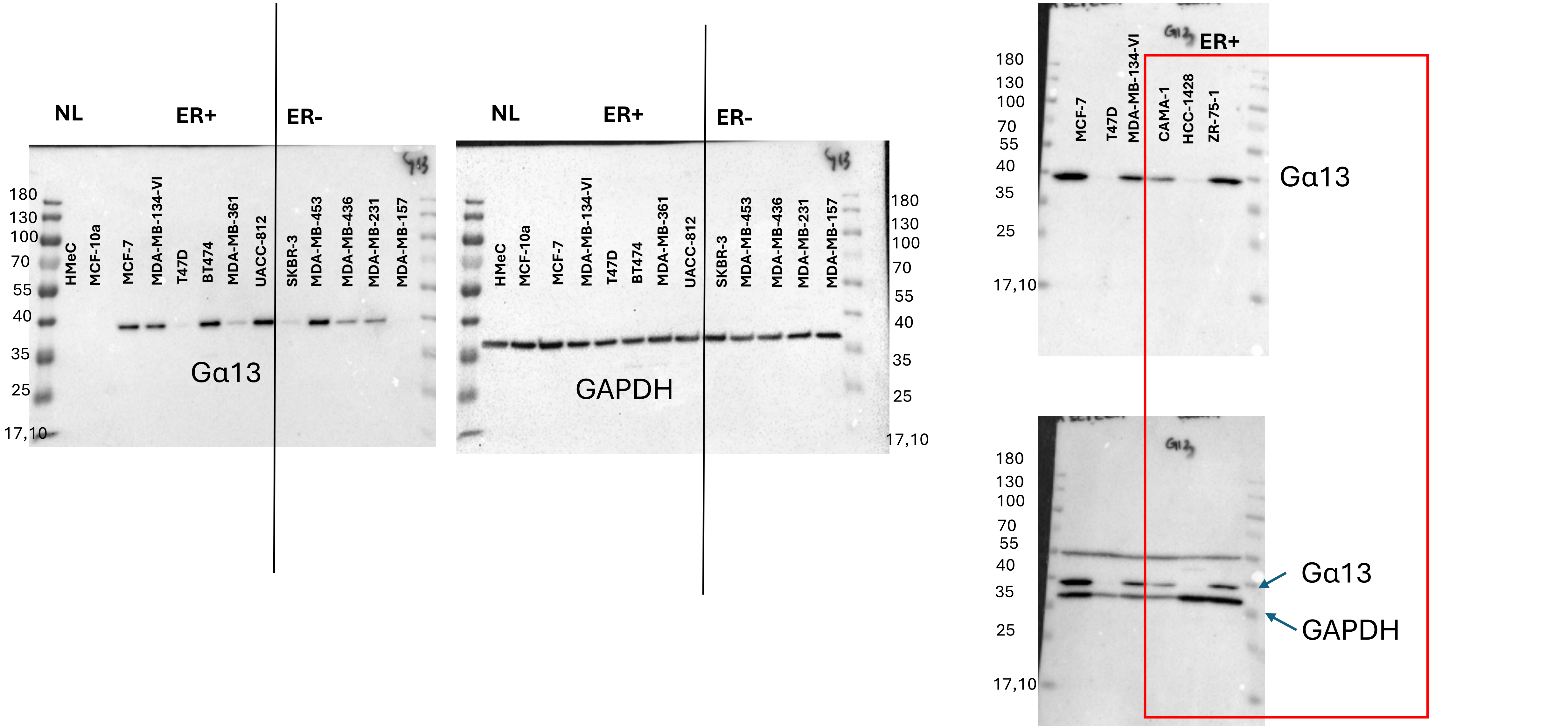

Fig 2A

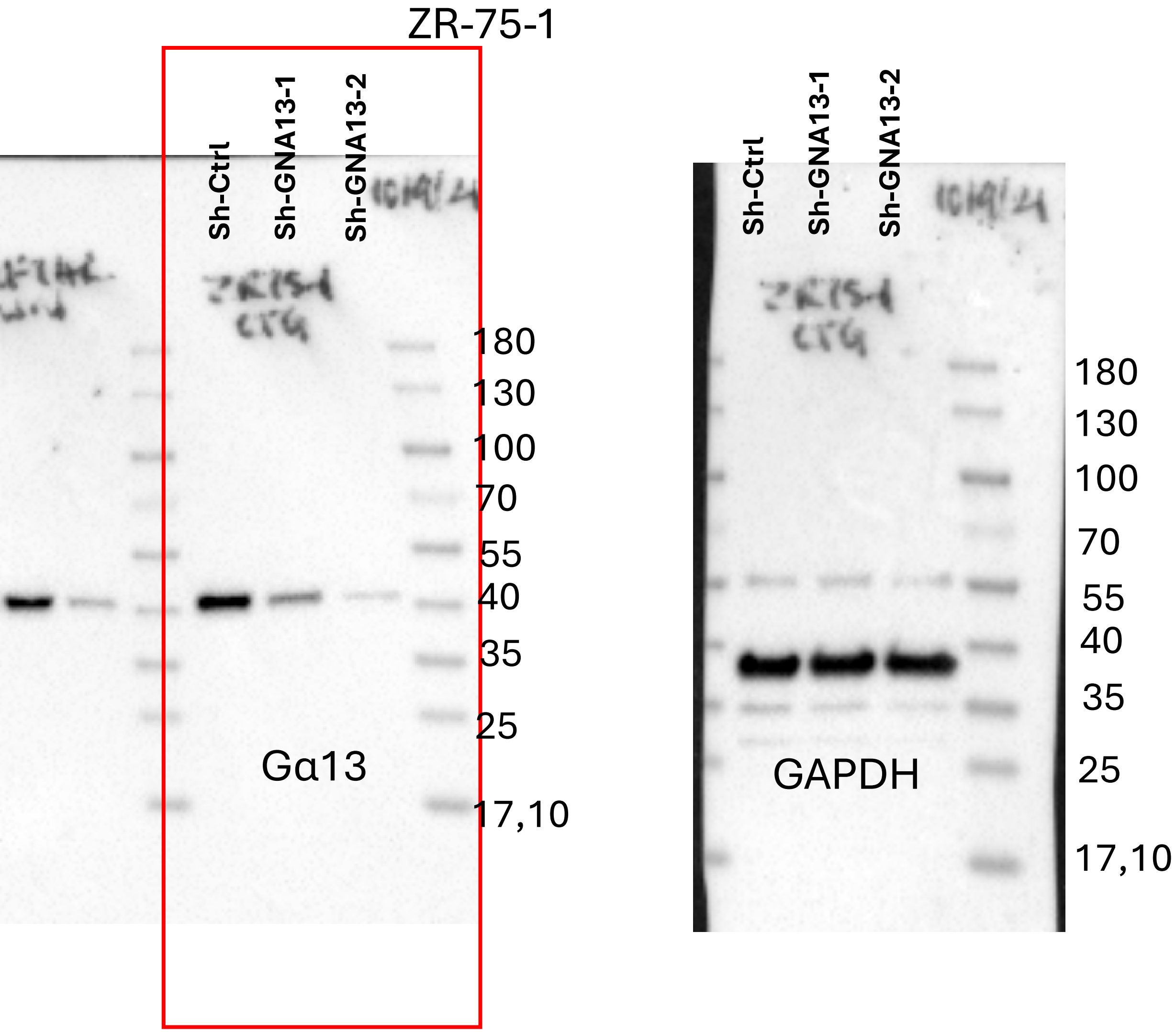
ZR-75-1
Sh-Ctrl
Sh-GNA13-1
Sh-GNA13-2
180
130
100
70
55
40
35
25
17,10
Gα13
Sh-Ctrl
Sh-GNA13-1
Sh-GNA13-2
180
130
100
70
55
40
35
25
17,10
GAPDH

Fig 2B

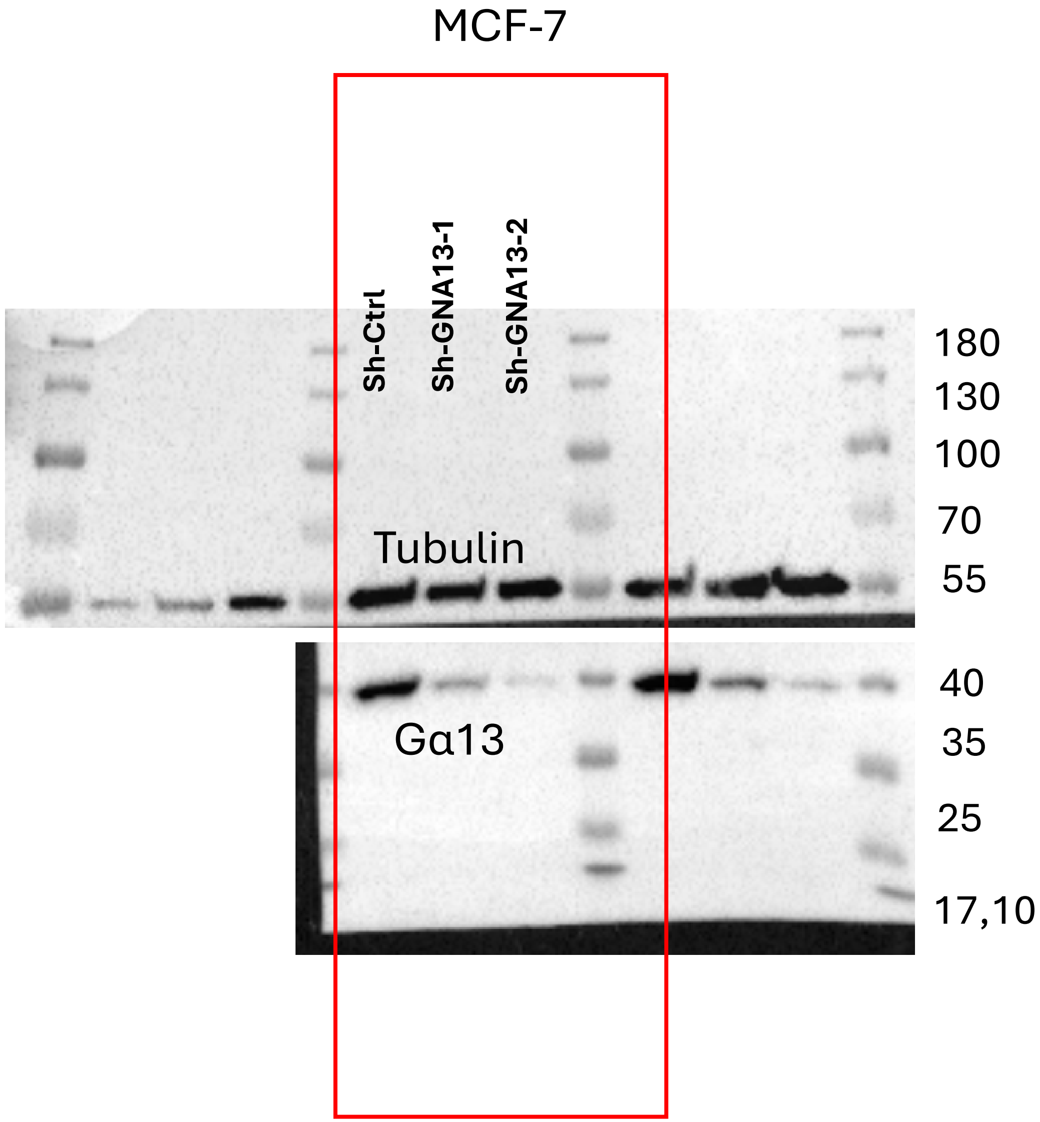
MCF-7
Sh-Ctrl
Sh-GNA13-1
Sh-GNA13-2
180
130
100
70
Tubulin
55
40
Gα13
35
25
17,10

Fig 2C

Lane 1 Ladder
Lane 2 shCtrl
Lane 3 shCtrl+GNA13
Lane 4 shGNA13
Lane 5 sh GNA13+GNA13

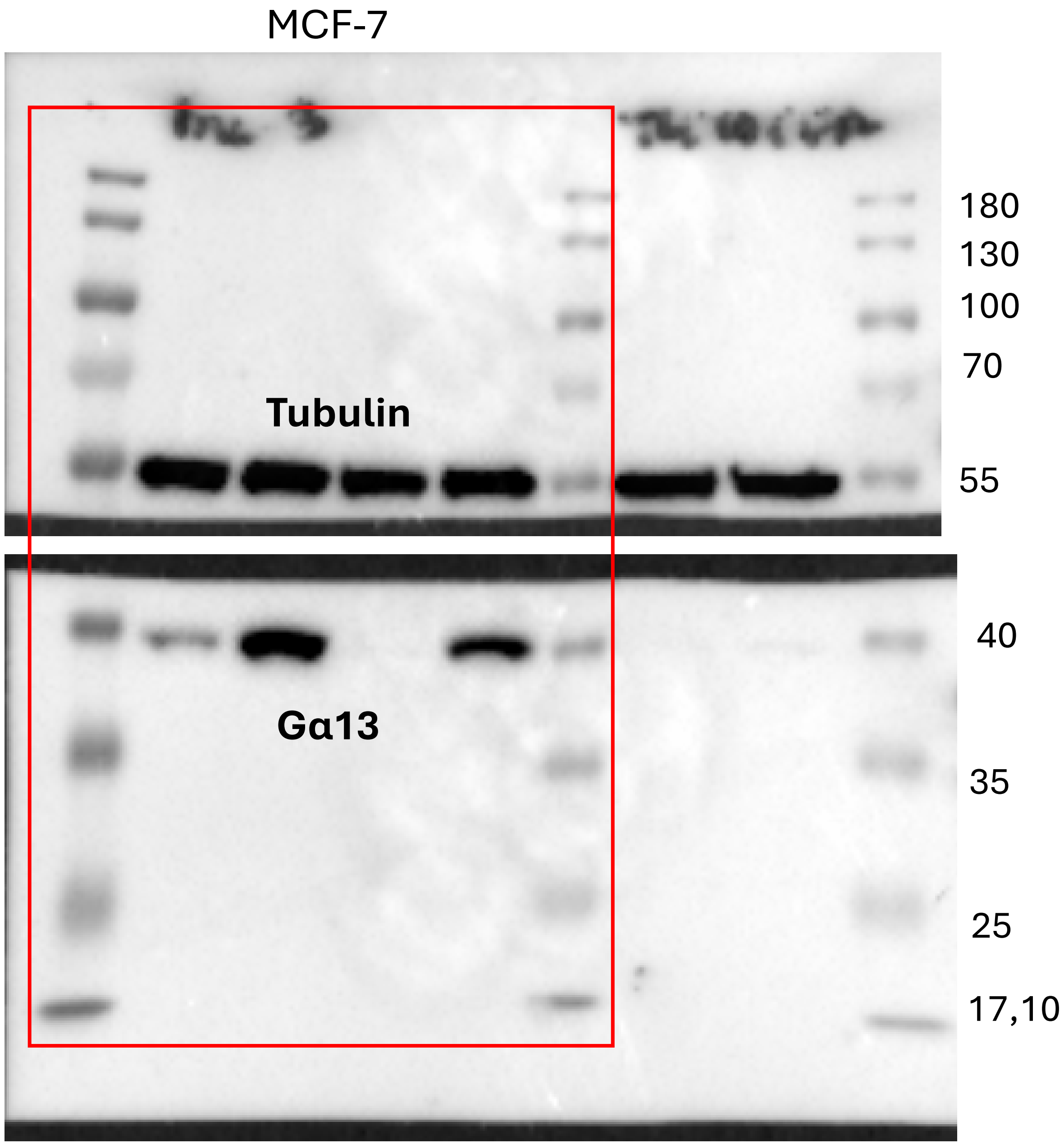

Fig 2D

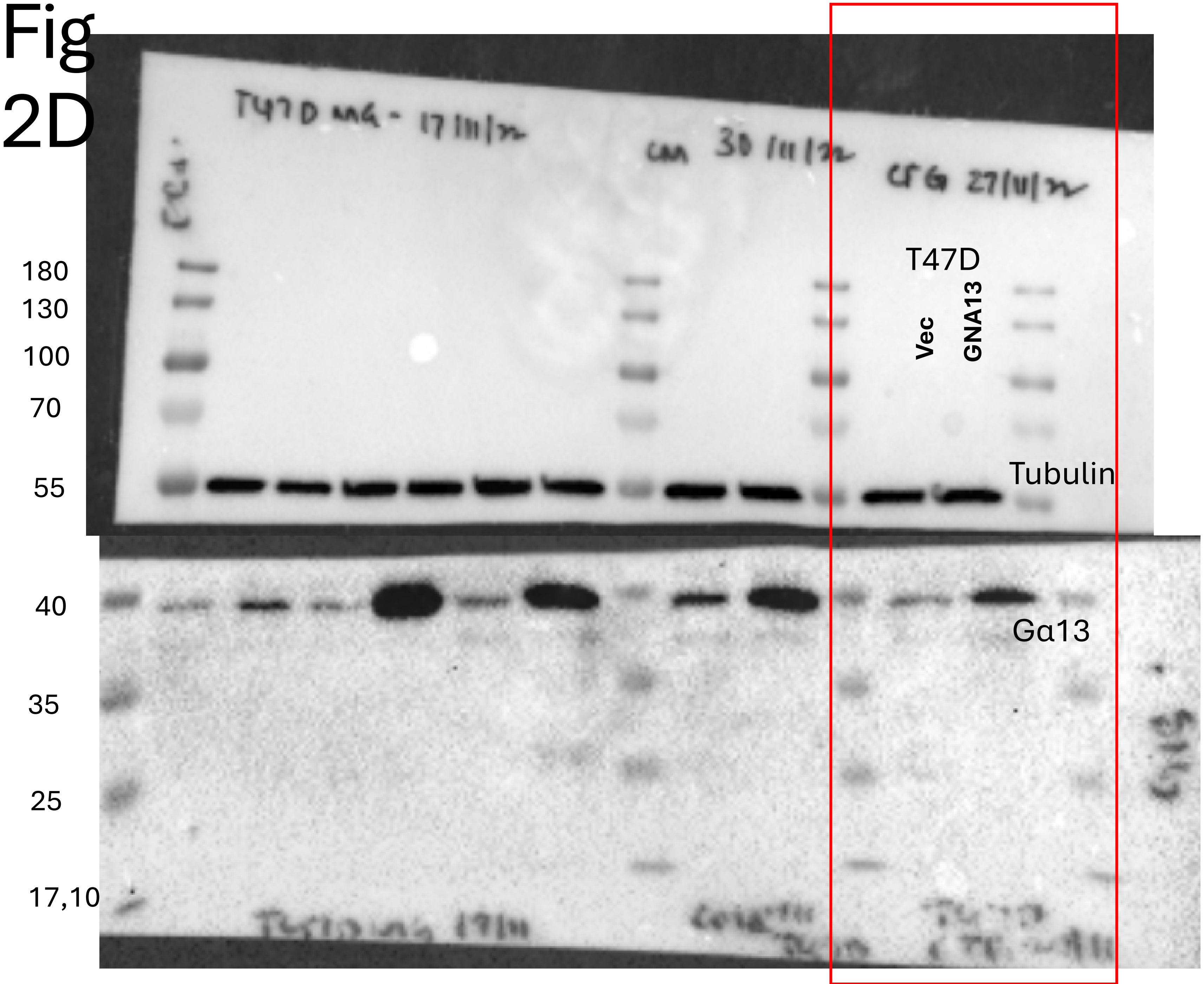

Fig 2E

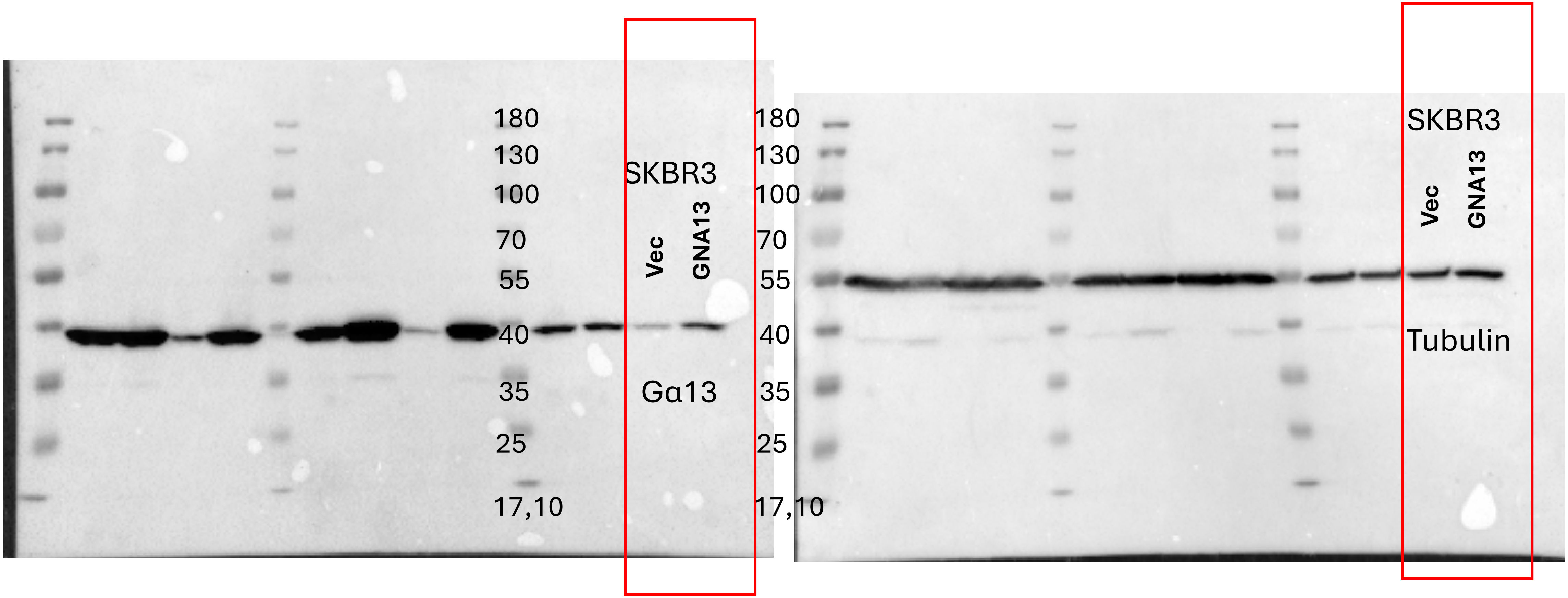
180
130
100
70
55
40
35
25
17,10
SKBR3
Vec
GNA13
Gα13
180
130
100
70
55
40
35
25
17,10
SKBR3
Vec
GNA13
Tubulin

Fig 2F

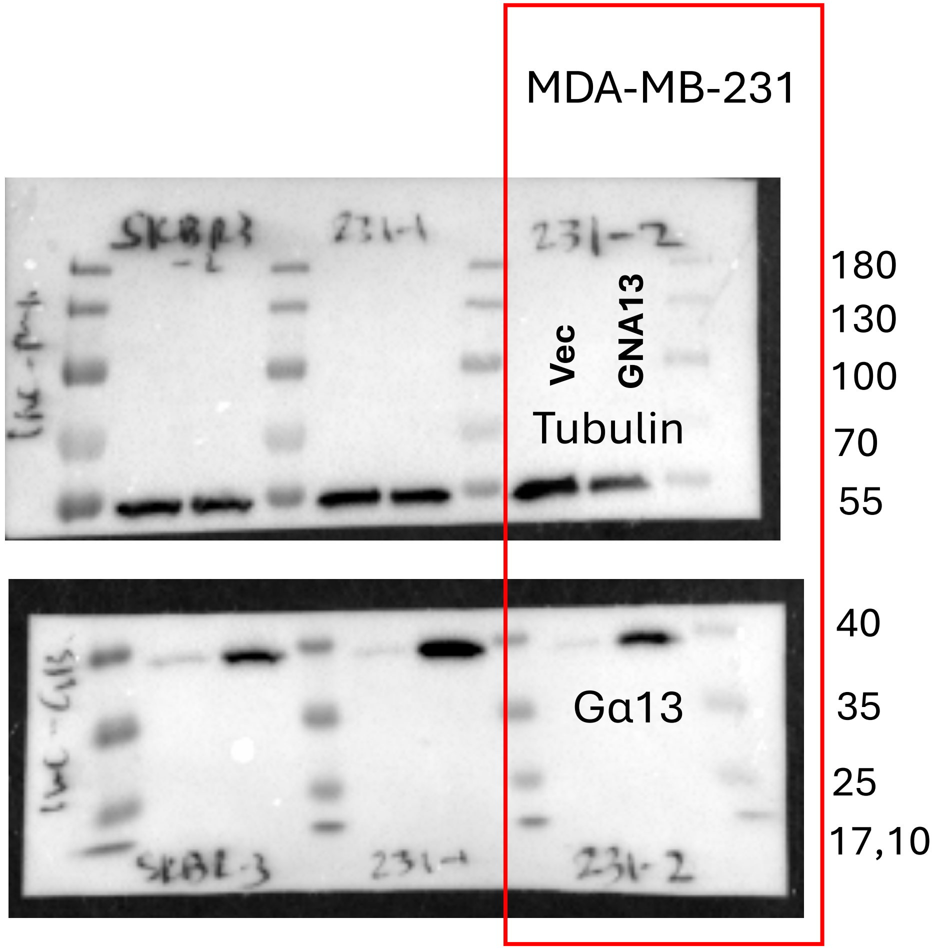
MDA-MB-231
Vec
GNA13
Tubulin
Gα13
180
130
100
70
55
40
35
25
17,10

Fig 5B

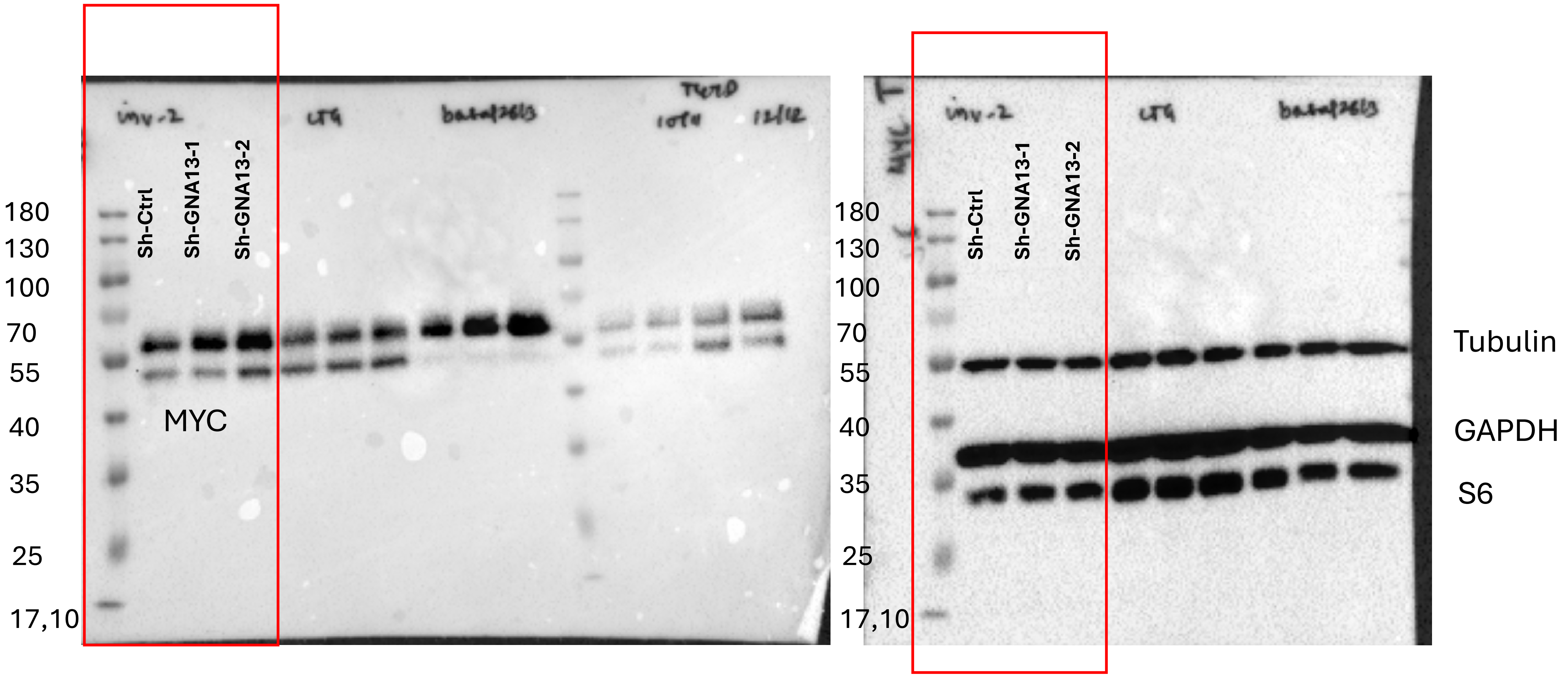

180
130
100
70
55
40
35
25
17,10
Sh-Ctrl
Sh-GNA13-1
Sh-GNA13-2
MYC
Tubulin
GAPDH
S6

Fig 5D

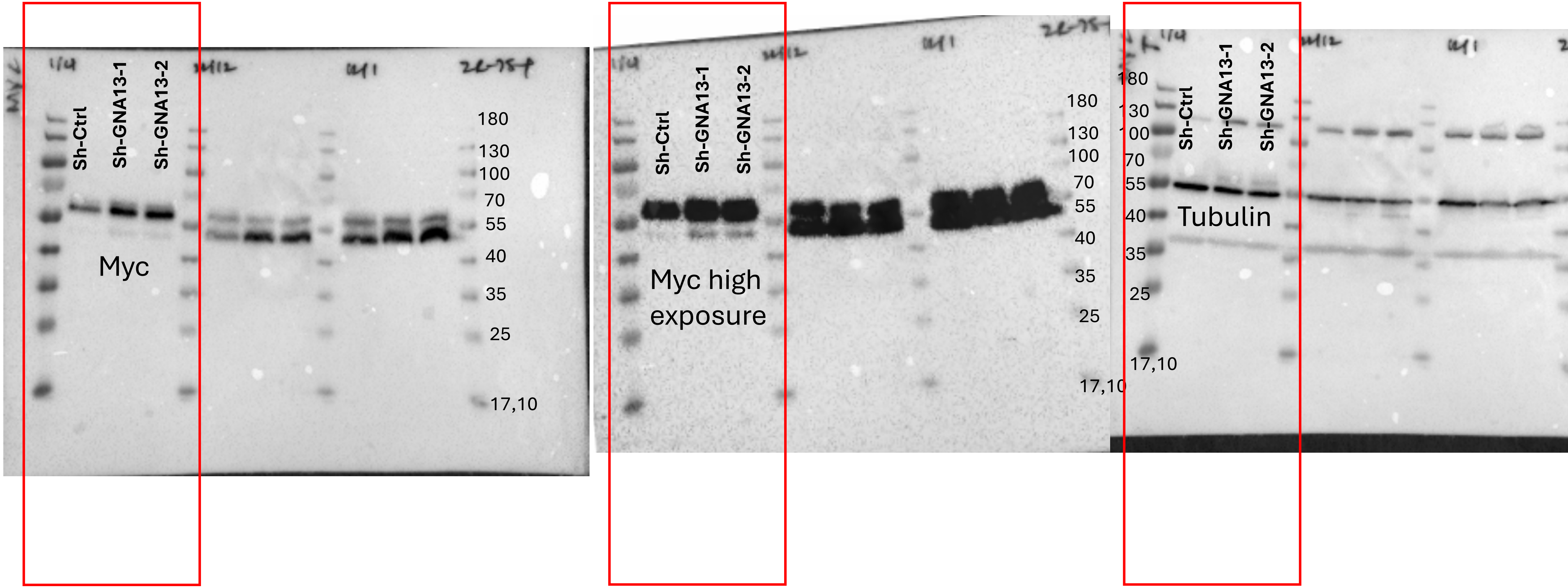

Sh-Ctrl
Sh-GNA13-1
Sh-GNA13-2
180
130
100
70
55
40
35
25
17,10
Myc
Myc high exposure
Tubulin

siMock- transfection controls not used in the final interpretation of data

Fig 5E

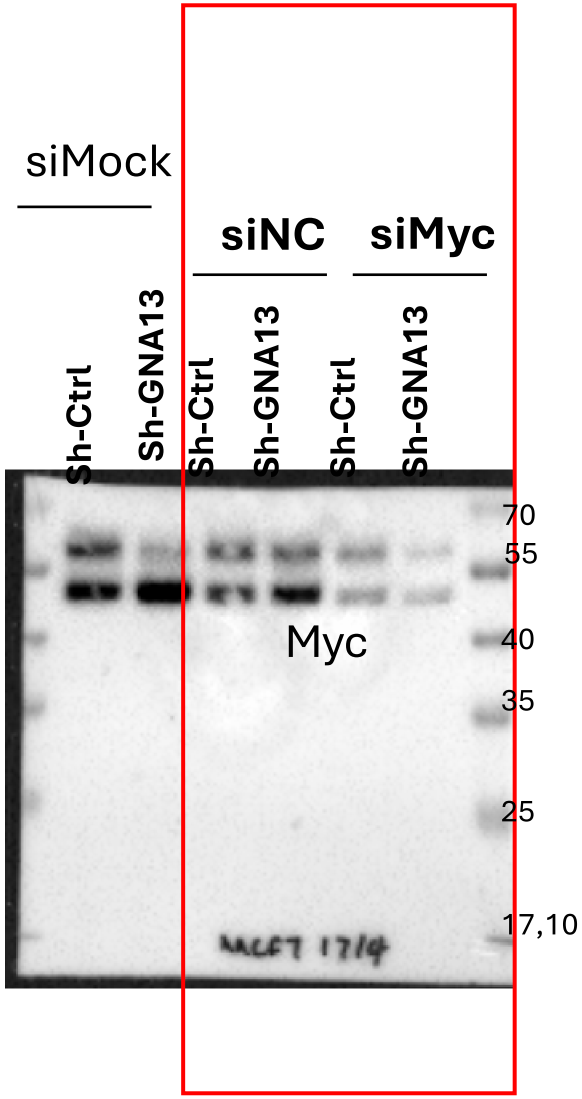

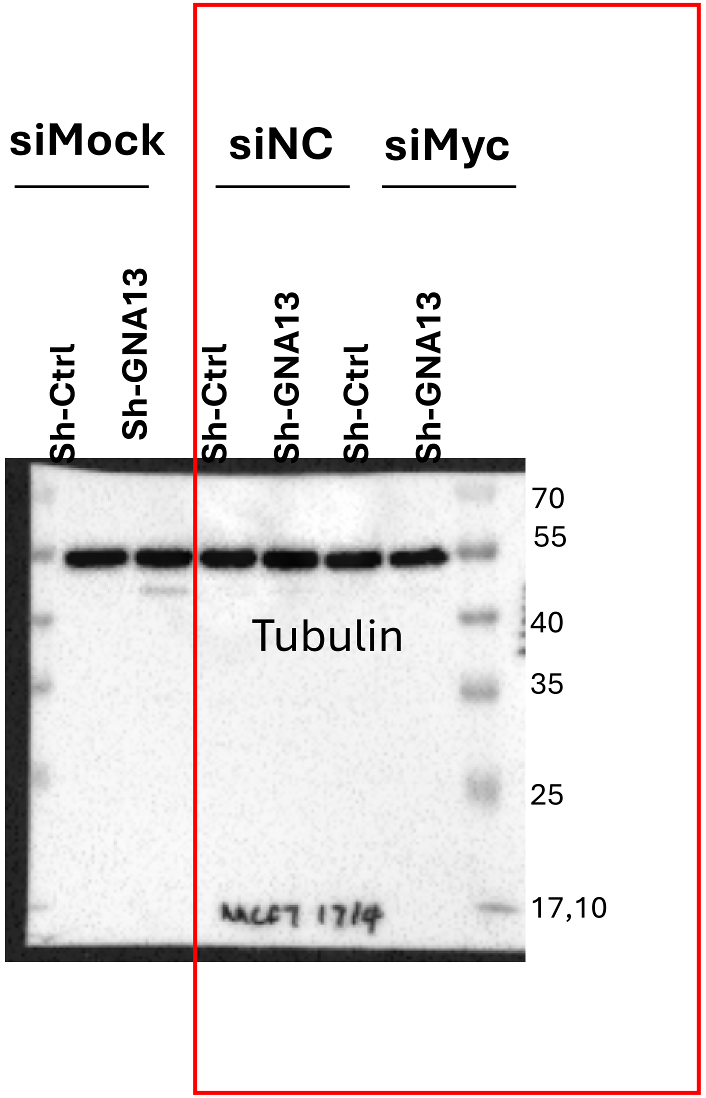

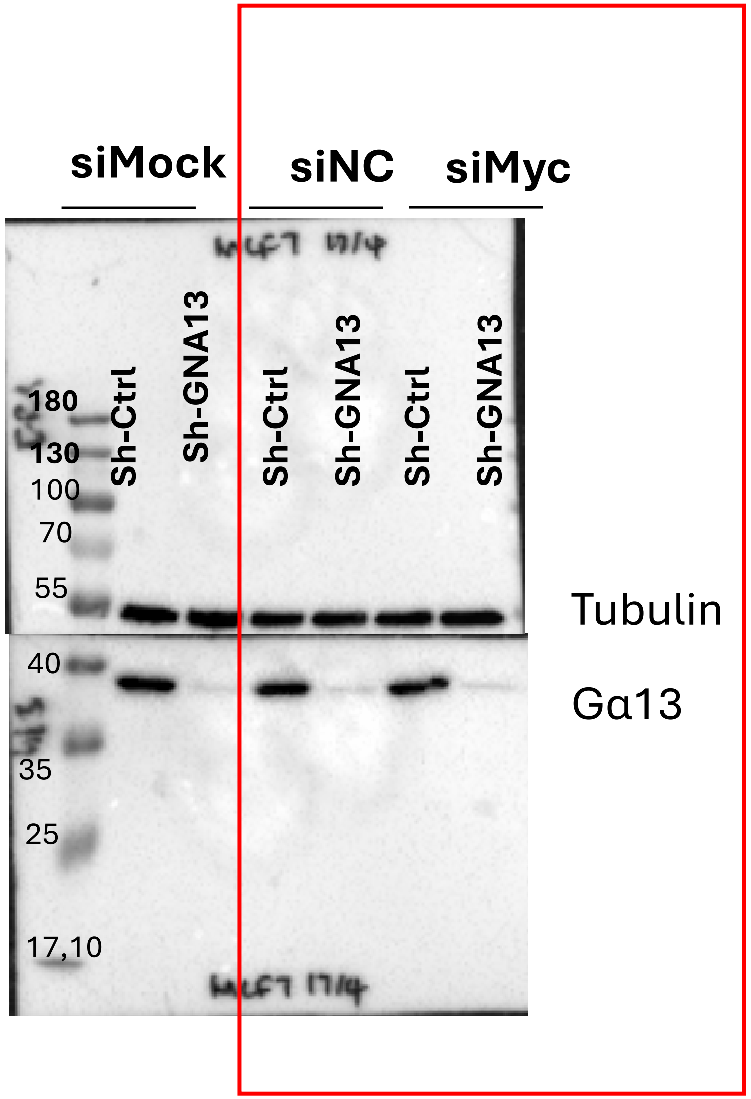

siMock- transfection controls not used in the final interpretation of data

Fig 5G

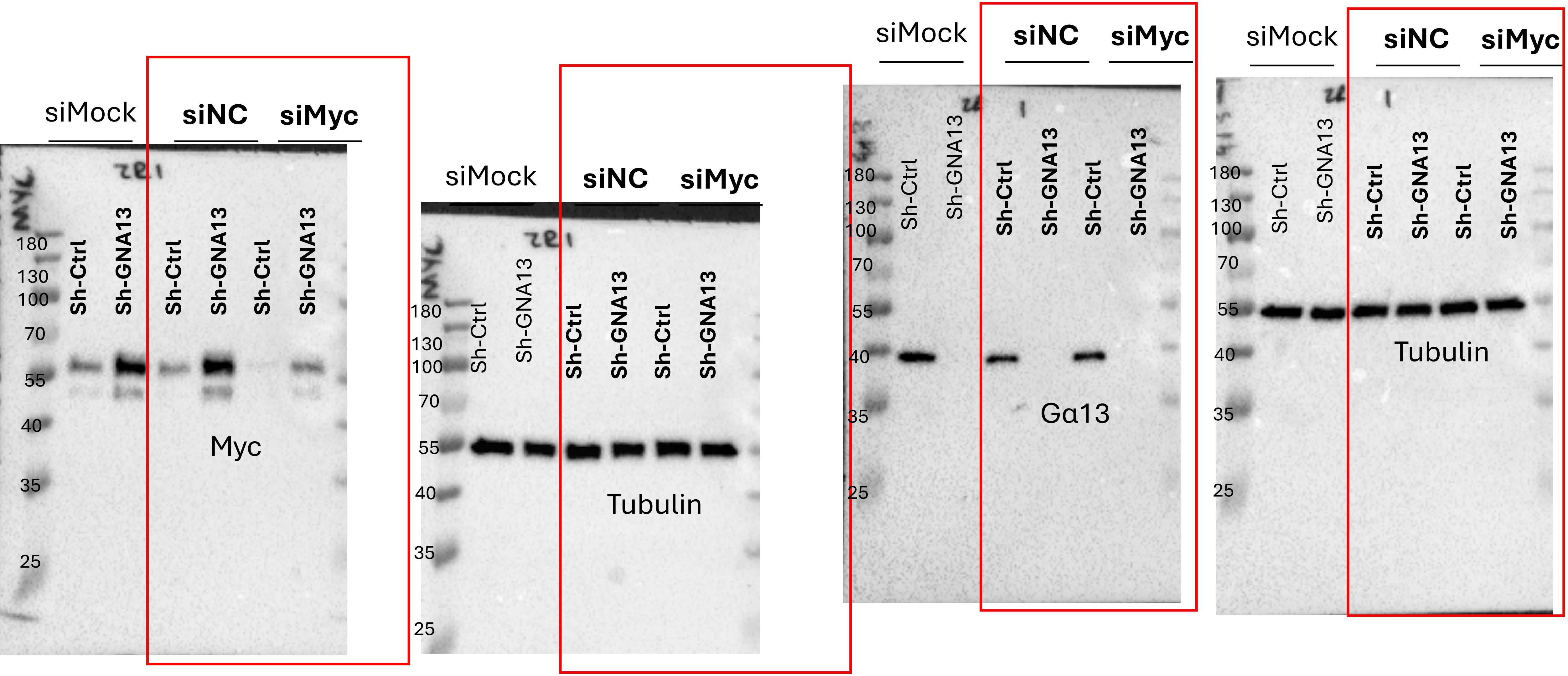

Fig 6A

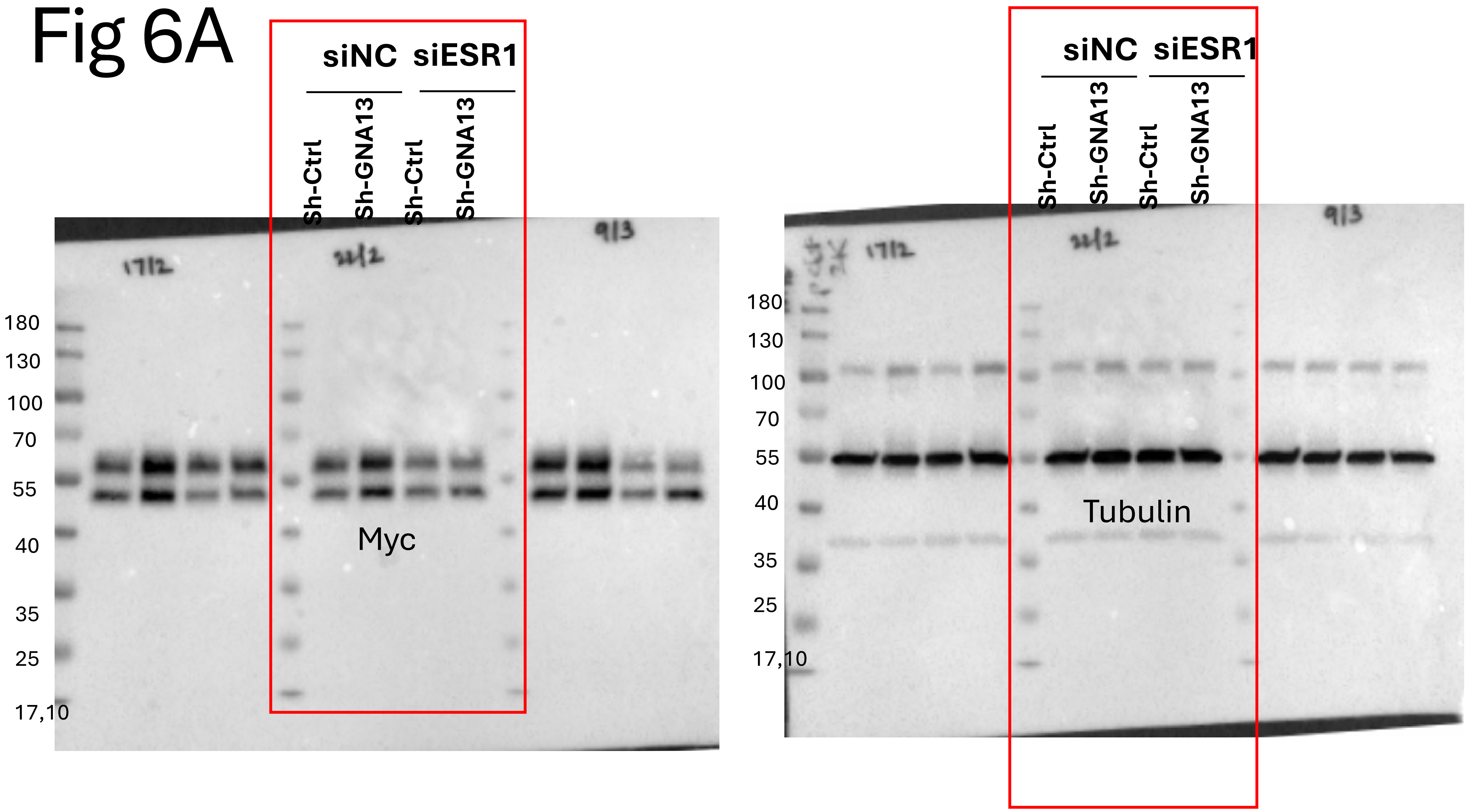
siNC
siESR1
Sh-Ctrl
Sh-GNA13
Sh-Ctrl
Sh-GNA13
180
130
100
70
55
40
35
25
17,10
Myc
siNC
siESR1
Sh-Ctrl
Sh-GNA13
Sh-Ctrl
Sh-GNA13
180
130
100
70
55
40
35
25
17,10
Tubulin

Fig 6A

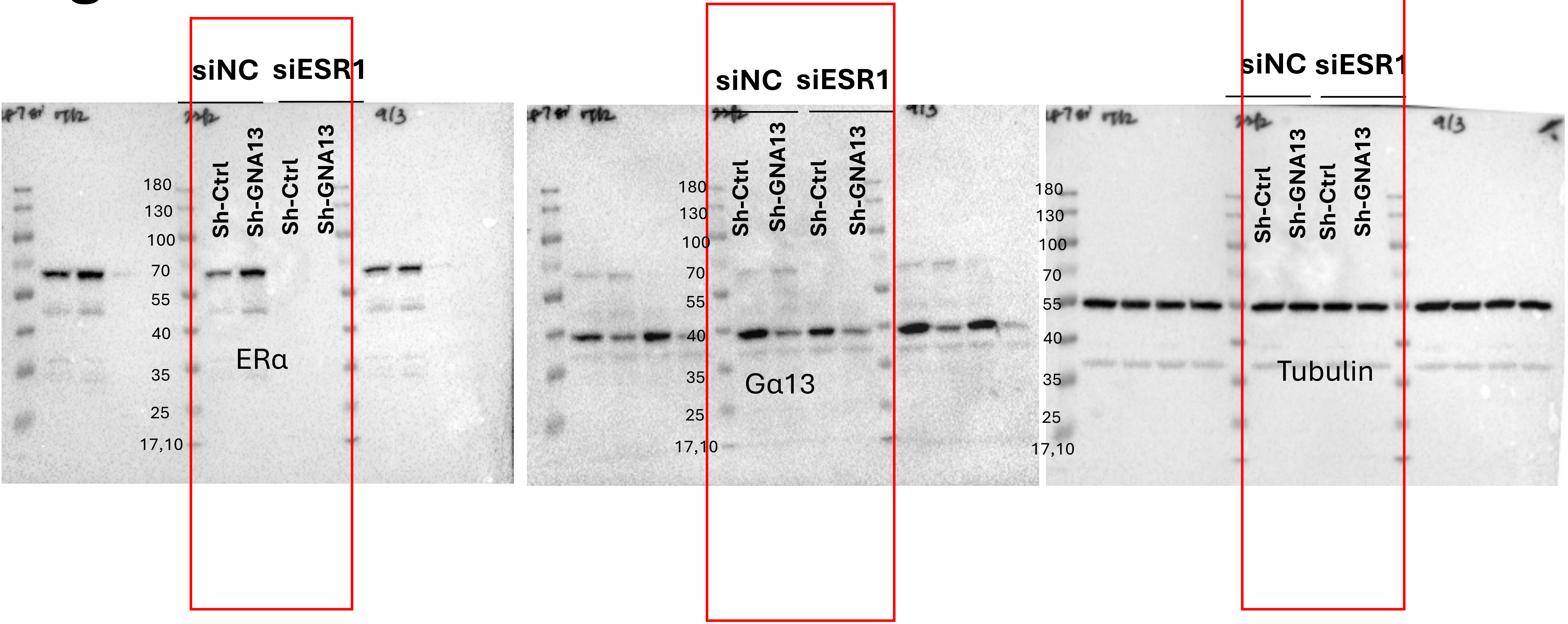
siNC
siESR1
Sh-Ctrl
Sh-GNA13
Sh-Ctrl
Sh-GNA13
180
130
100
70
55
40
35
25
17,10
ERα
siNC
siESR1
Sh-Ctrl
Sh-GNA13
Sh-Ctrl
Sh-GNA13
180
130
100
70
55
40
35
25
17,10
Gα13
siNC
siESR1
Sh-Ctrl
Sh-GNA13
Sh-Ctrl
Sh-GNA13
180
130
100
70
55
40
35
25
17,10
Tubulin

Fig 6B

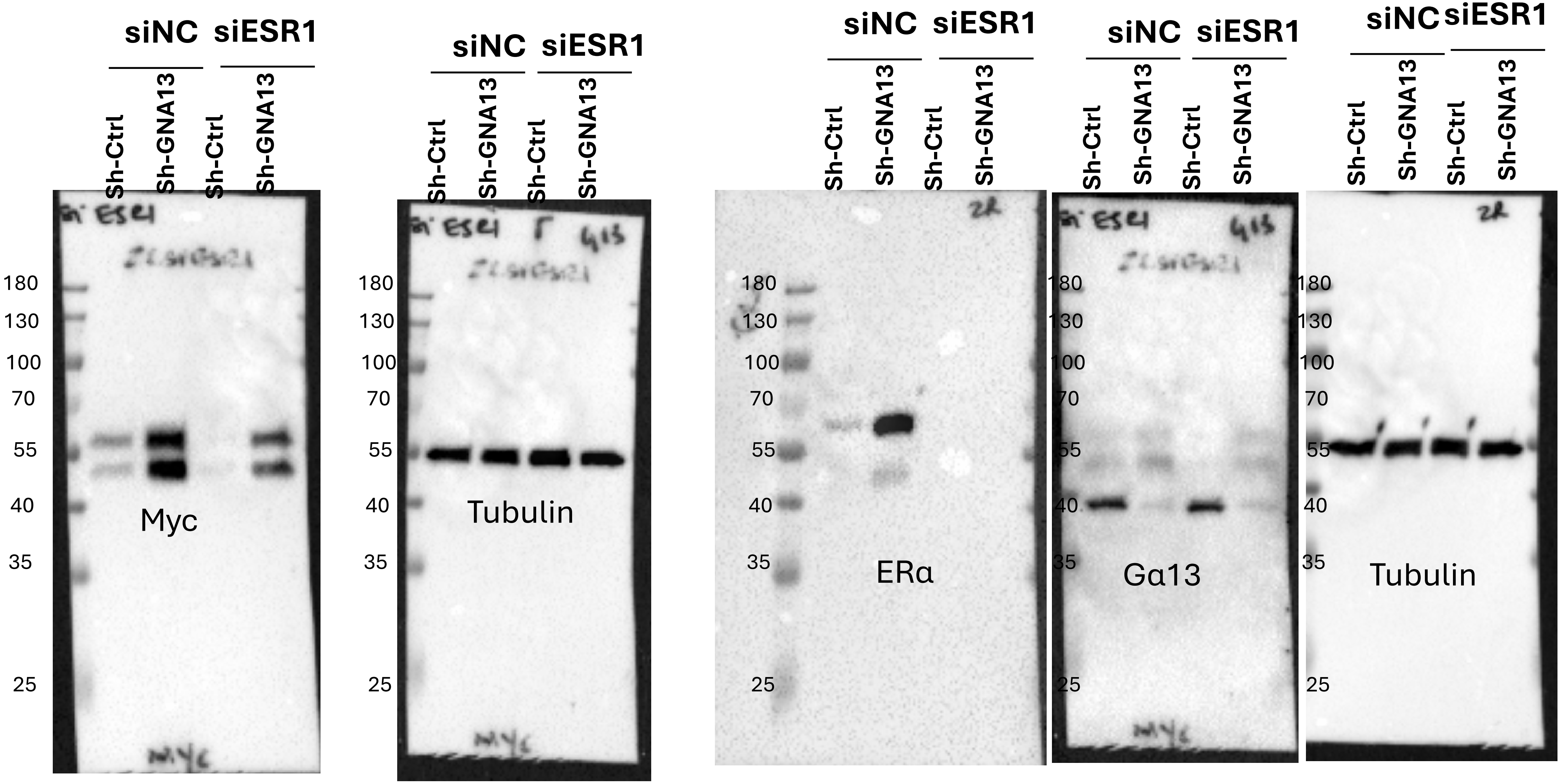
siNC
siESR1
Sh-Ctrl
Sh-GNA13
Sh-Ctrl
Sh-GNA13
180
130
100
70
55
40
35
25
Myc
Tubulin
ERα
Gα13
Tubulin

Fig 6C

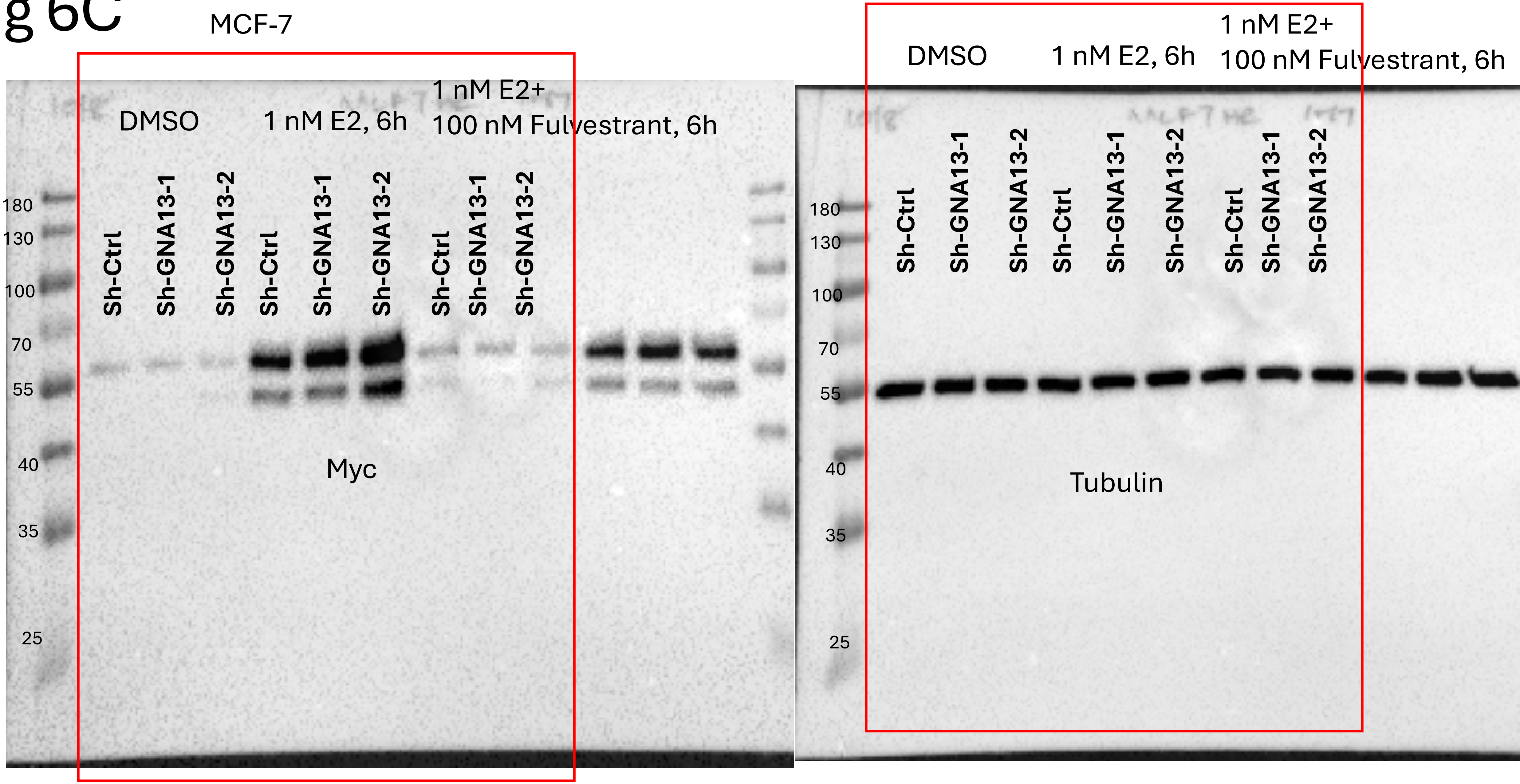
MCF-7
DMSO
1 nM E2, 6h
1 nM E2+
100 nM Fulvestrant, 6h
180
130
100
70
55
40
35
25
Sh-Ctrl
Sh-GNA13-1
Sh-GNA13-2
Sh-Ctrl
Sh-GNA13-1
Sh-GNA13-2
Sh-Ctrl
Sh-GNA13-1
Sh-GNA13-2
Myc
DMSO
1 nM E2, 6h
1 nM E2+
100 nM Fulvestrant, 6h
180
130
100
70
55
40
35
25
Sh-Ctrl
Sh-GNA13-1
Sh-GNA13-2
Sh-Ctrl
Sh-GNA13-1
Sh-GNA13-2
Sh-Ctrl
Sh-GNA13-1
Sh-GNA13-2
Tubulin

Fig 6C

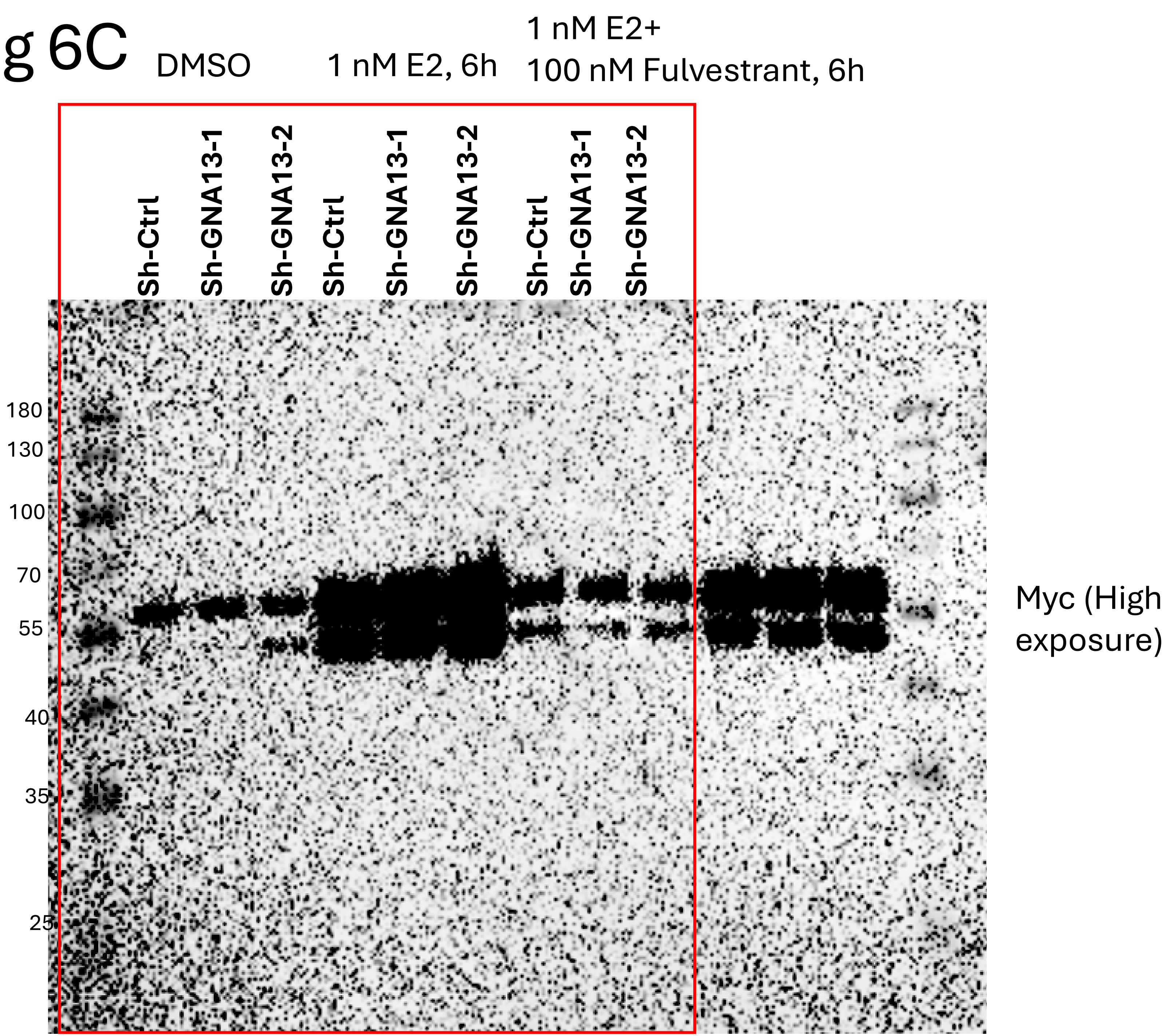
DMSO
1 nM E2, 6h
1 nM E2+
100 nM Fulvestrant, 6h
Sh-Ctrl
Sh-GNA13-1
Sh-GNA13-2
Sh-Ctrl
Sh-GNA13-1
Sh-GNA13-2
Sh-Ctrl
Sh-GNA13-1
Sh-GNA13-2
180
130
100
70
55
40
35
25
Myc (High exposure)

Fig 6C

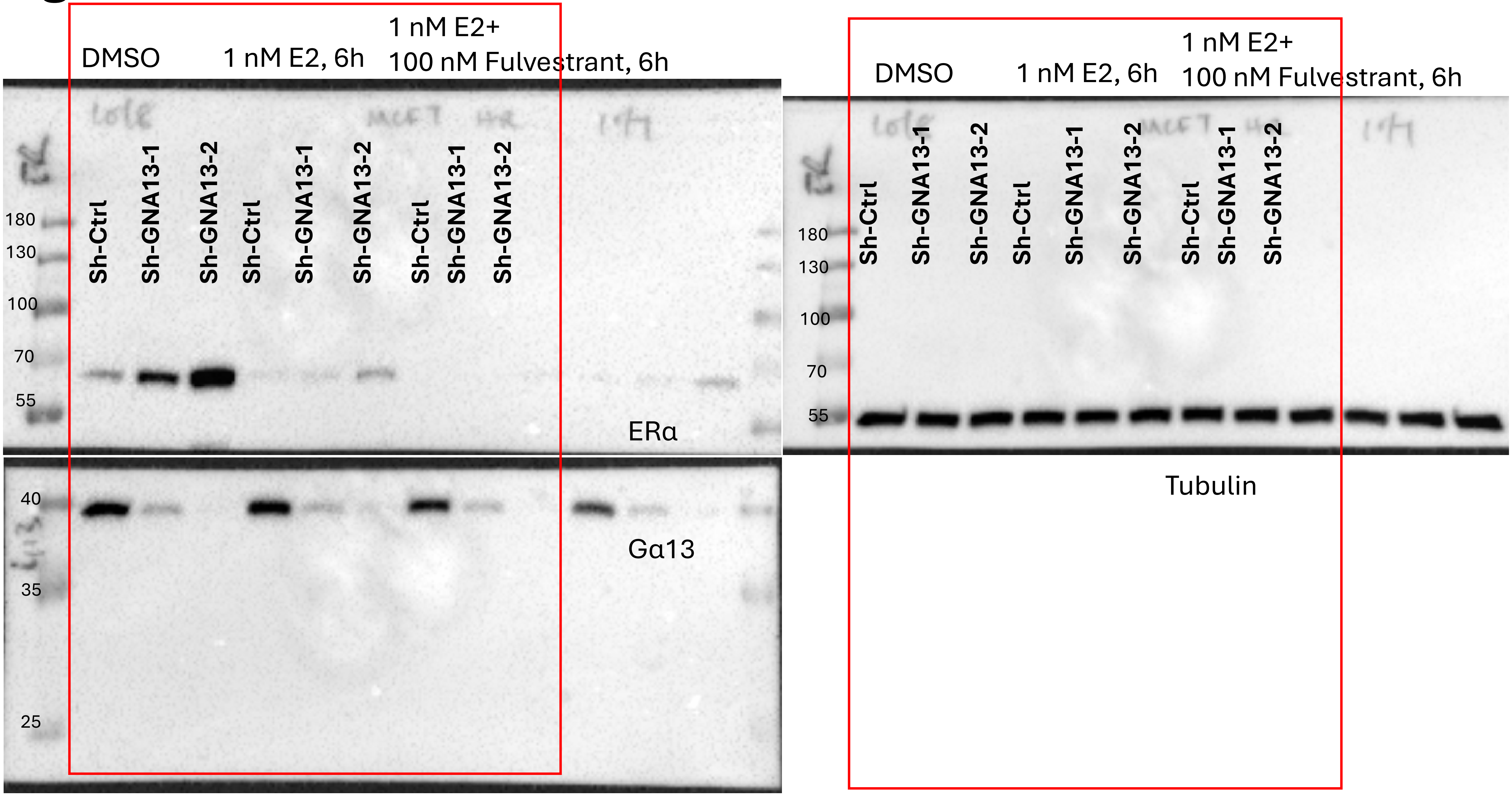

DMSO
1 nM E2, 6h
1 nM E2+
100 nM Fulvestrant, 6h
Sh-Ctrl
Sh-GNA13-1
Sh-GNA13-2
Sh-Ctrl
Sh-GNA13-1
Sh-GNA13-2
Sh-Ctrl
Sh-GNA13-1
Sh-GNA13-2
180
130
100
70
55
ERα
40
35
25
Gα13
DMSO
1 nM E2, 6h
1 nM E2+
100 nM Fulvestrant, 6h
Sh-Ctrl
Sh-GNA13-1
Sh-GNA13-2
Sh-Ctrl
Sh-GNA13-1
Sh-GNA13-2
Sh-Ctrl
Sh-GNA13-1
Sh-GNA13-2
180
130
100
70
55
Tubulin

Fig 6C

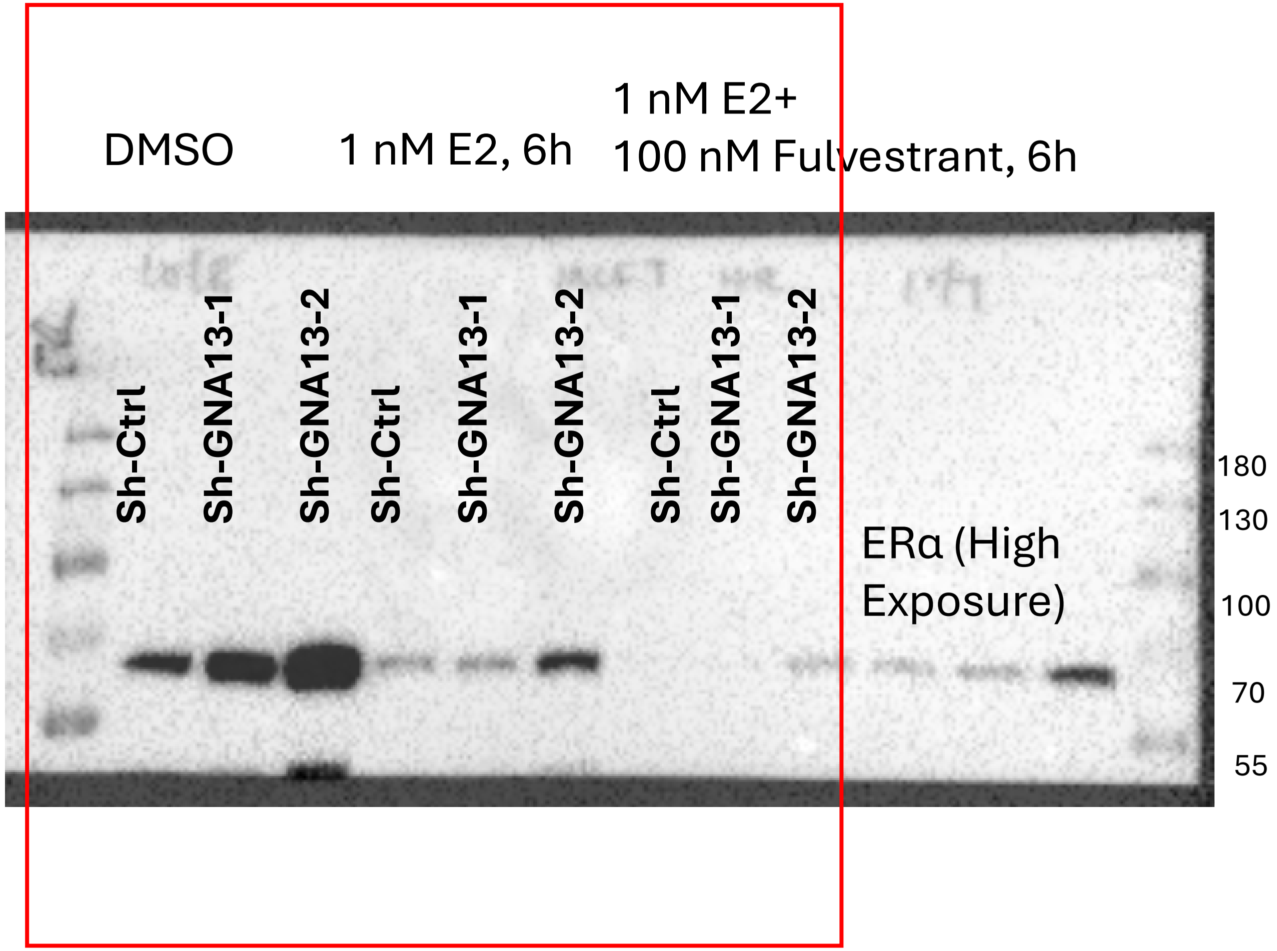

DMSO
1 nM E2, 6h
1 nM E2+
100 nM Fulvestrant, 6h
Sh-Ctrl
Sh-GNA13-1
Sh-GNA13-2
Sh-Ctrl
Sh-GNA13-1
Sh-GNA13-2
Sh-Ctrl
Sh-GNA13-1
Sh-GNA13-2
ERα (High Exposure)
180
130
100
70
55

Fig 6D

ZR-75-1 HR

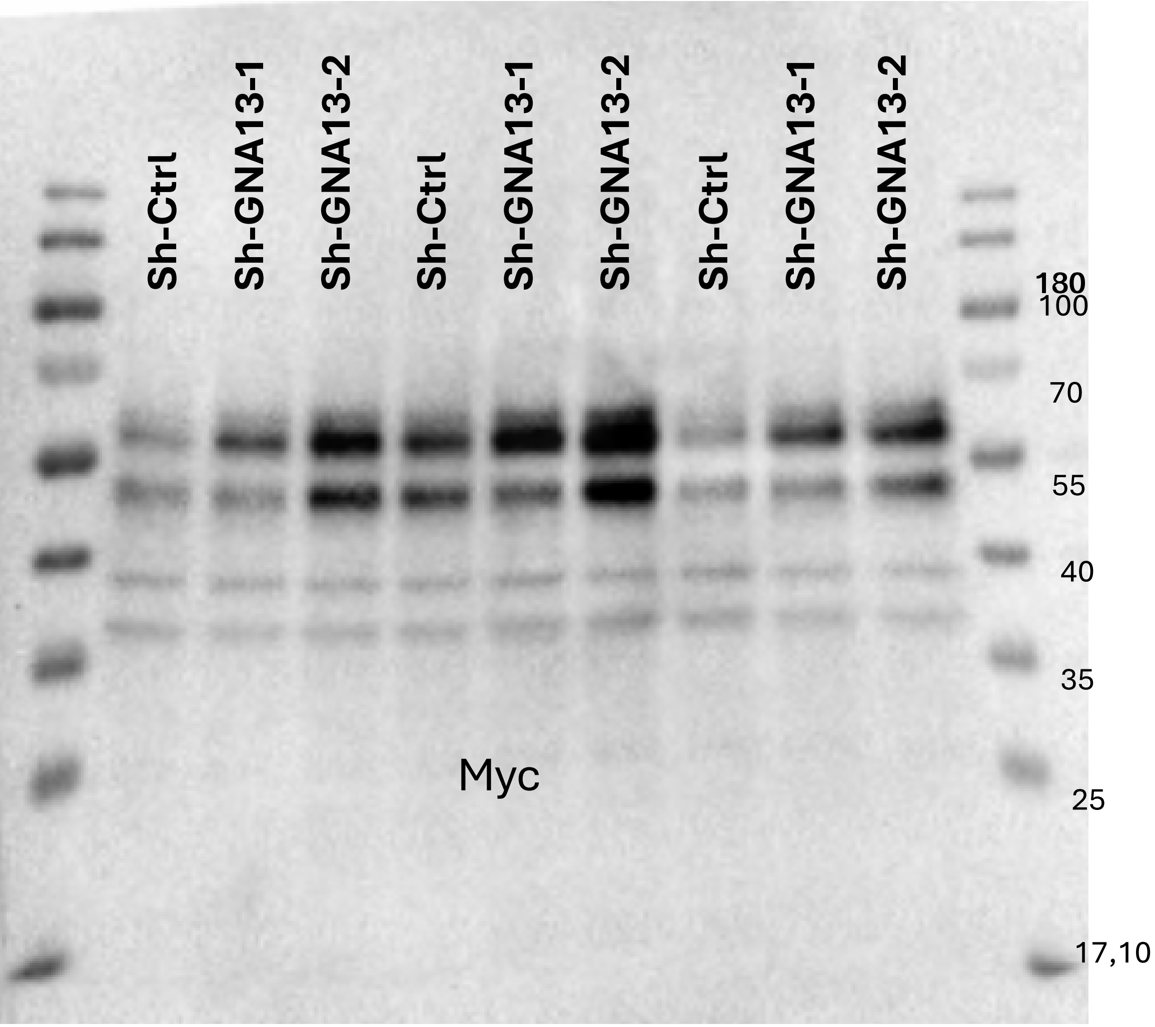

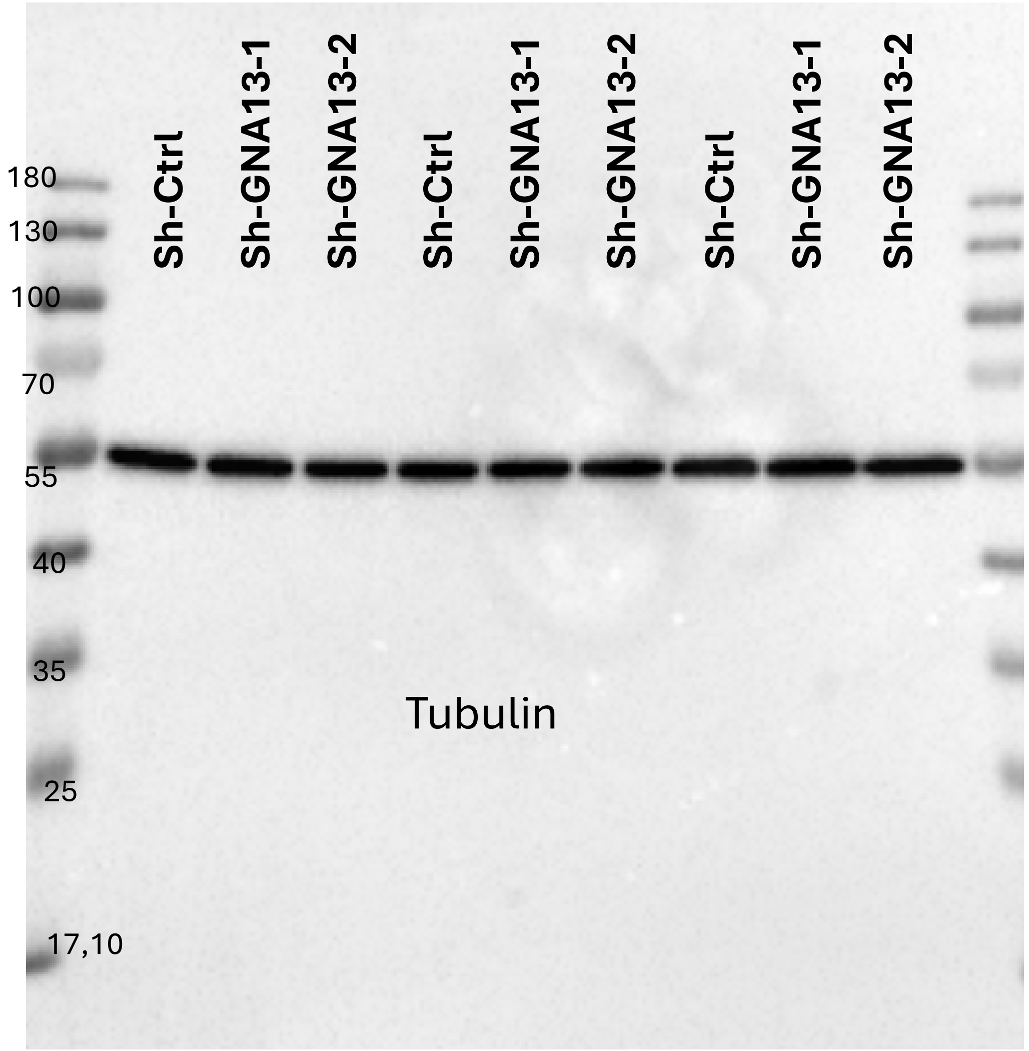

Fig 6D

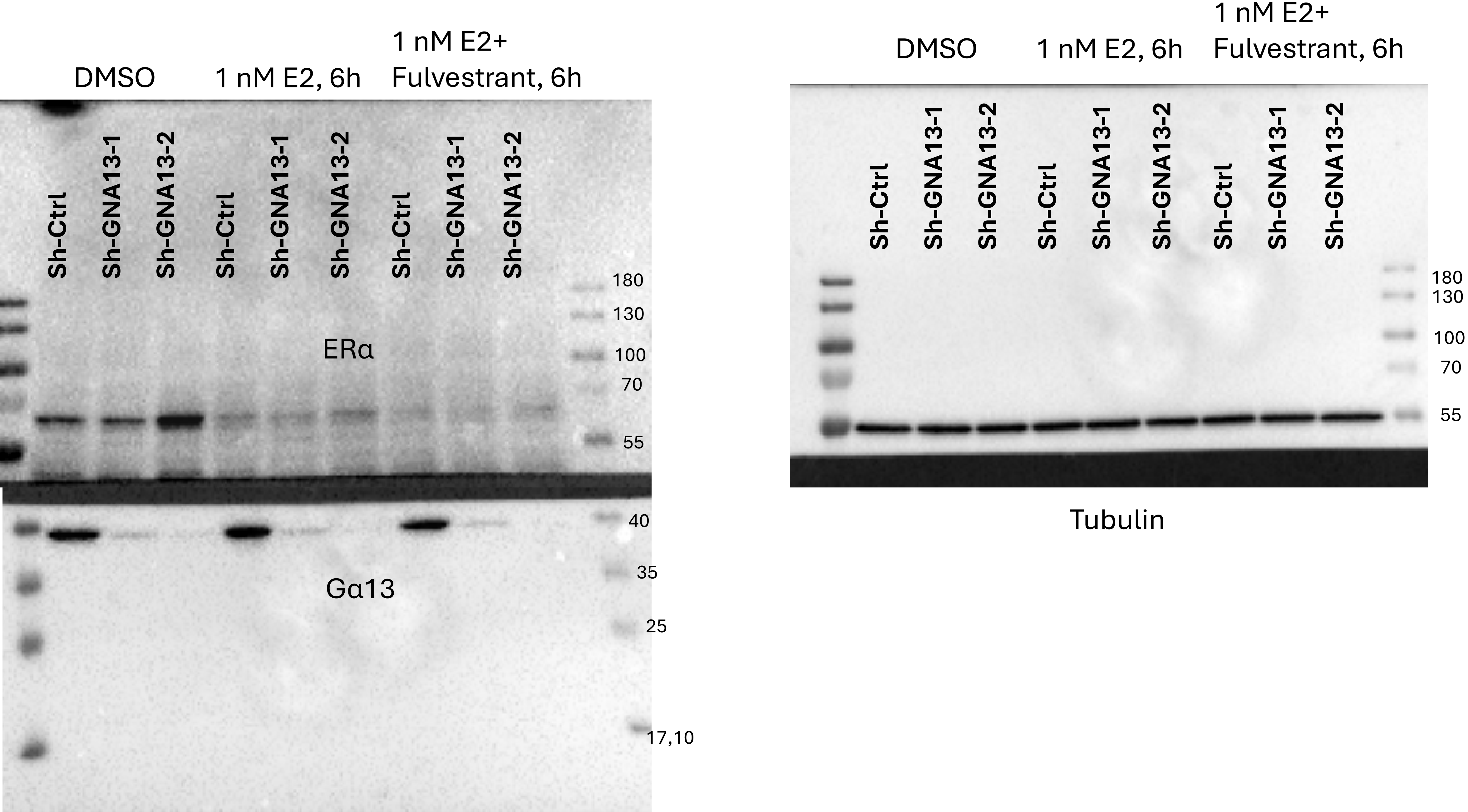
DMSO
1 nM E2, 6h
1 nM E2+ Fulvestrant, 6h
Sh-Ctrl
Sh-GNA13-1
Sh-GNA13-2
Sh-Ctrl
Sh-GNA13-1
Sh-GNA13-2
Sh-Ctrl
Sh-GNA13-1
Sh-GNA13-2
180
130
100
70
55
ERα
40
35
25
17,10
Gα13
DMSO
1 nM E2, 6h
1 nM E2+ Fulvestrant, 6h
Sh-Ctrl
Sh-GNA13-1
Sh-GNA13-2
Sh-Ctrl
Sh-GNA13-1
Sh-GNA13-2
Sh-Ctrl
Sh-GNA13-1
Sh-GNA13-2
180
130
100
70
55
Tubulin

Fig 6E

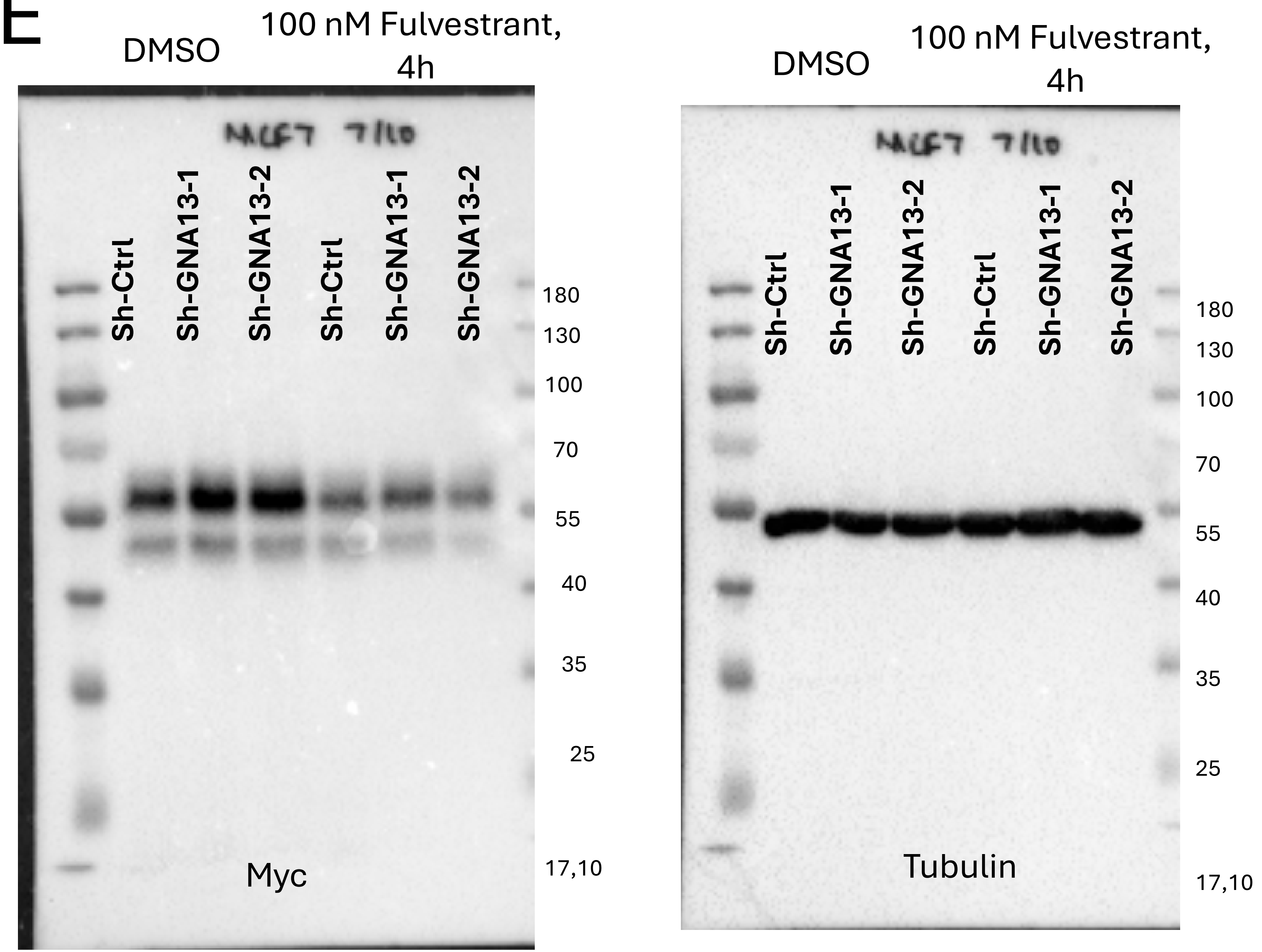
DMSO
100 nM Fulvestrant, 4h
MCF7 7/10
Sh-Ctrl
Sh-GNA13-1
Sh-GNA13-2
Sh-Ctrl
Sh-GNA13-1
Sh-GNA13-2
180
130
100
70
55
40
35
25
17,10
Myc
DMSO
100 nM Fulvestrant, 4h
MCF7 7/10
Sh-Ctrl
Sh-GNA13-1
Sh-GNA13-2
Sh-Ctrl
Sh-GNA13-1
Sh-GNA13-2
180
130
100
70
55
40
35
25
17,10
Tubulin

Fig 6E

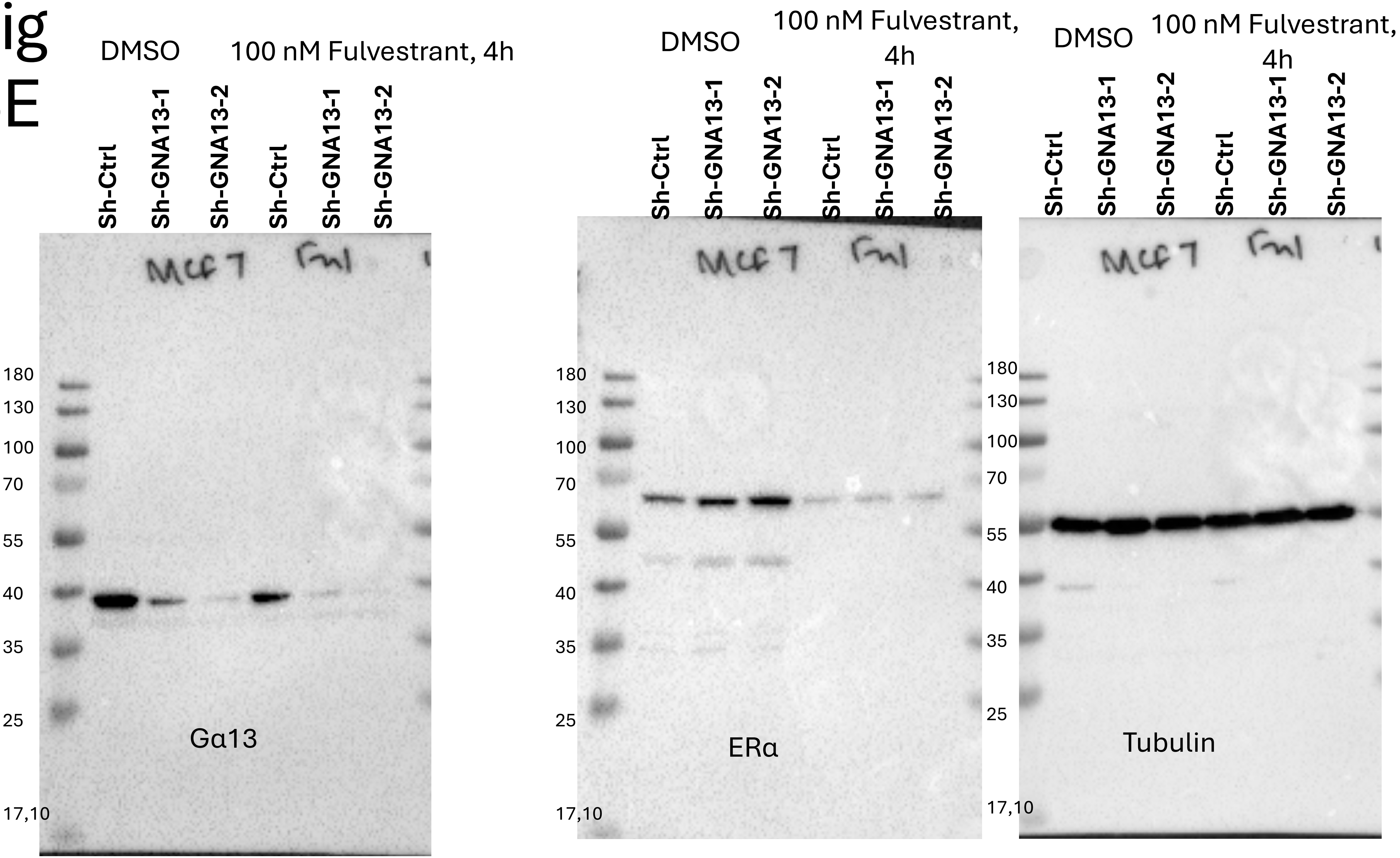

DMSO
100 nM Fulvestrant, 4h
Sh-Ctrl
Sh-GNA13-1
Sh-GNA13-2
Sh-Ctrl
Sh-GNA13-1
Sh-GNA13-2
180
130
100
70
55
40
35
25
17,10
Gα13
DMSO
100 nM Fulvestrant, 4h
Sh-Ctrl
Sh-GNA13-1
Sh-GNA13-2
Sh-Ctrl
Sh-GNA13-1
Sh-GNA13-2
180
130
100
70
55
40
35
25
17,10
ERα
DMSO
100 nM Fulvestrant, 4h
Sh-Ctrl
Sh-GNA13-1
Sh-GNA13-2
Sh-Ctrl
Sh-GNA13-1
Sh-GNA13-2
180
130
100
70
55
40
35
25
17,10
Tubulin

Fig 6F

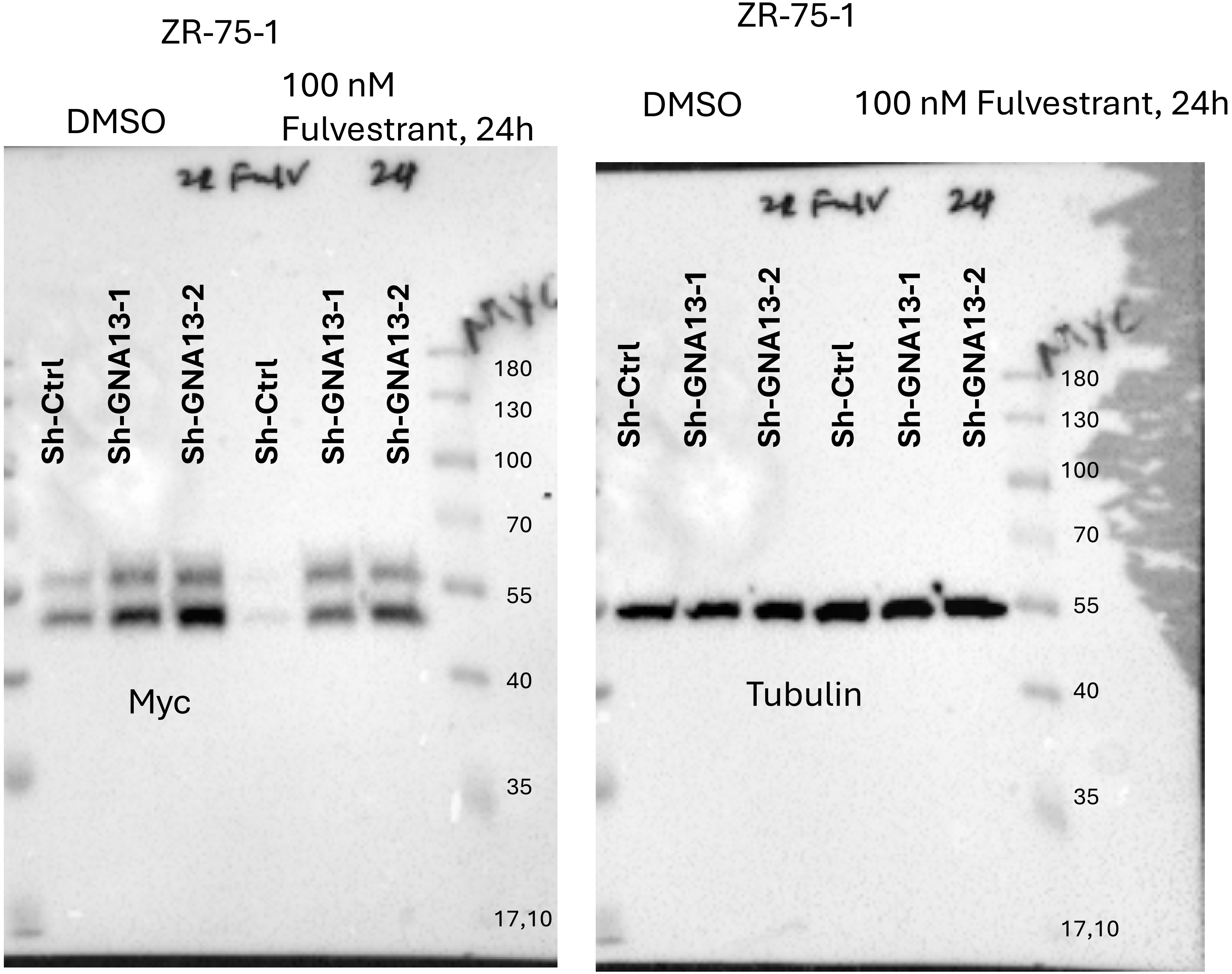
ZR-75-1
DMSO
100 nM Fulvestrant, 24h
Sh-Ctrl
Sh-GNA13-1
Sh-GNA13-2
Sh-Ctrl
Sh-GNA13-1
Sh-GNA13-2
180
130
100
70
55
40
35
17,10
Myc
ZR-75-1
DMSO
100 nM Fulvestrant, 24h
Sh-Ctrl
Sh-GNA13-1
Sh-GNA13-2
Sh-Ctrl
Sh-GNA13-1
Sh-GNA13-2
180
130
100
70
55
40
35
17,10
Tubulin

ZR-75-1

Fig 6F

ZR-75-1

DMSO 100 nM Fulvestrant, 24h

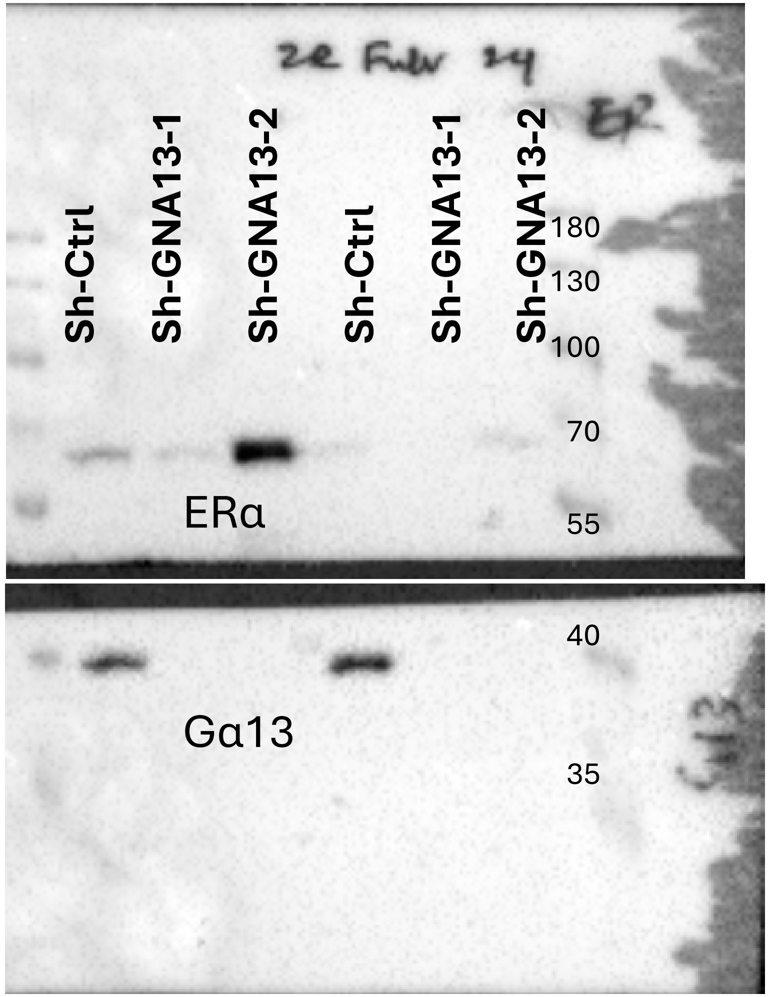

ZR-75-1

DMSO 100 nM Fulvestrant, 24h

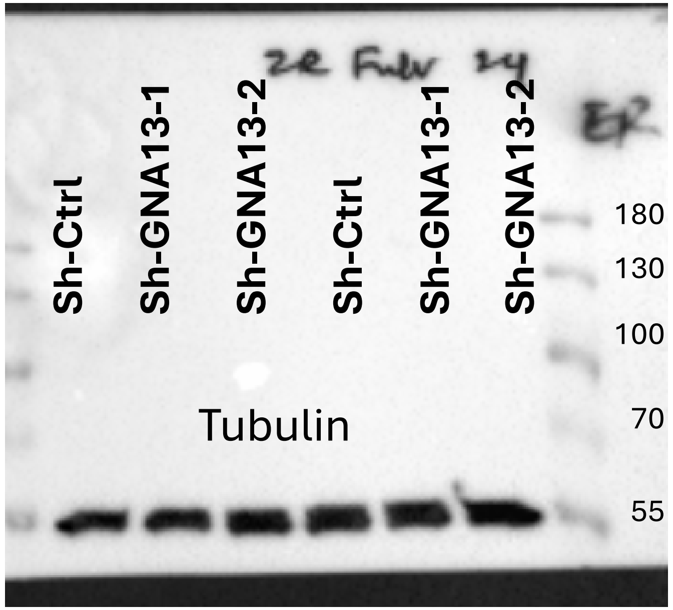

Fig S3B

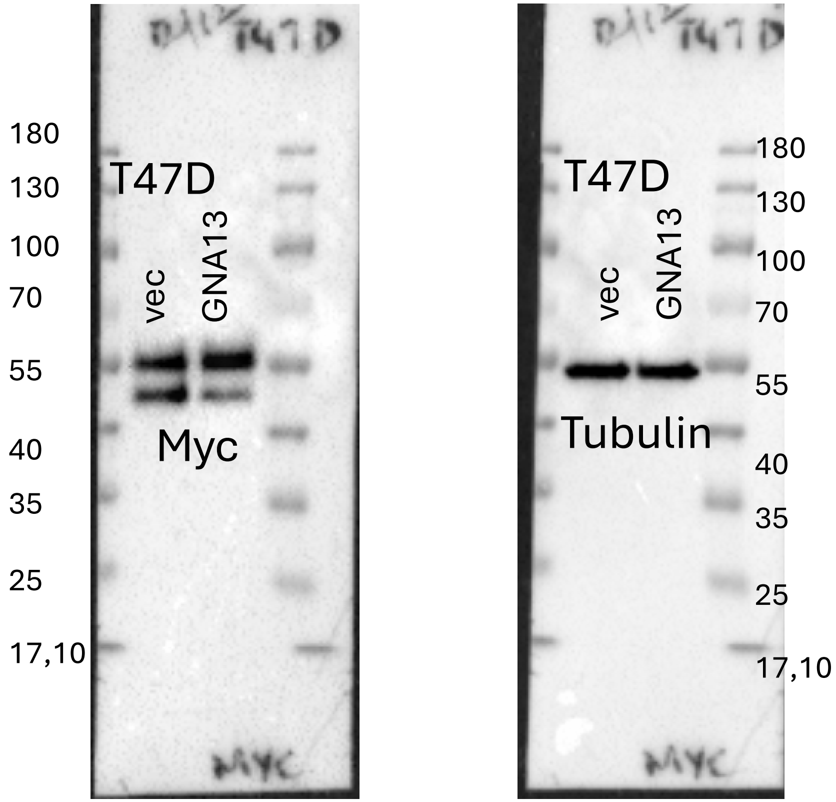
180
130
100
70
55
40
35
25
17,10
T47D
vec
GNA13
Myc
T47D
vec
GNA13
Tubulin
180
130
100
70
55
40
35
25
17,10
